# Diversity and distribution of reptiles in Romania

**DOI:** 10.3897/zookeys.341.5502

**Published:** 2013-10-08

**Authors:** Dan Cogălniceanu, Laurentiu Rozylowicz, Paul Székely, Ciprian Samoilă, Florina Stănescu, Marian Tudor, Diana Székely, Ruben Iosif

**Affiliations:** 1 University Ovidius Constanţa, Faculty of Natural Sciences and Agricultural Sciences, Al. Universităţii nr. 1, corp B, 900470, Constanţa, Romania; 2 University of Bucharest, Center for Environmental Research and Impact Studies, Bd. N. Bălcescu nr. 1, 010041, Bucharest, Romania

**Keywords:** Reptilia, species distribution, species range, biodiversity data, species richness, rarity

## Abstract

The reptile fauna of Romania comprises 23 species, out of which 12 species reach here the limit of their geographic range. We compiled and updated a national database of the reptile species occurrences from a variety of sources including our own field surveys, personal communication from specialists, museum collections and the scientific literature. The occurrence records were georeferenced and stored in a geodatabase for additional analysis of their spatial patterns. The spatial analysis revealed a biased sampling effort concentrated in various protected areas, and deficient in the vast agricultural areas of the southern part of Romania. The patterns of species richness showed a higher number of species in the warmer and drier regions, and a relatively low number of species in the rest of the country. Our database provides a starting point for further analyses, and represents a reliable tool for drafting conservation plans.

## Introduction

Reptiles are declining worldwide at an alarming rate (Gibbons et al. 2000, Böhm et al. 2013) and, along with amphibians, are considered among the most threatened vertebrate groups (Stuart et al. 2008, Hof et al. 2011). The decline of reptiles has been induced by a variety of threats such as habitat loss, degradation and fragmentation, pet trade, invasive species, pollution and diseases (Gibbons et al. 2000, Cox and Temple 2009). Currently, it is considered that climate changes severely affect reptiles (Böhm et al. 2013). Therefore, we need to assess the potential impacts of the forecasted global changes by assessing anticipated changes in the ecological niche of species. Although complex species distribution models have been developed, with some managing to predict the species ecological niches from just a few known localities (Phillips et al. 2006, Peterson et al. 2011), we still face difficulties to accurately assess the impact of these future threats. Among the limitations encountered when modeling ecological niches is the quality of the biological data used to train the models, which is the first condition to meet certain standards (Pineda and Lobo 2009). Data quality issues are triggered by use of improper spatial resolution, misidentification, missing data, and the lack of a proper sampling design (Araújo and Guisan 2006, Peterson et al. 2011). In this manner, it is mandatory to check for any bias in the sampling effort when mapping species distribution (e.g., Proess 2003, Loureiro et al. 2010).

Romania has five biogeographic regions (i.e., Alpine, Continental, Pannonian, Black Sea, and Steppic) out of the nine regions recognized by the European Union (Cogălniceanu and Cogălniceanu 2010, Iojă et al. 2010, Evans 2012). Romania still maintains a significant proportion of habitats with high conservation value, such as boreal coniferous forests, mesophilous, hygrophilous and xerothermic broadleaved forests, grassland and shrubby ecosystems. There is also a rich diversity of aquatic ecosystems including mountain springs and rivers, river floodplains, glacial lakes, coastal wetlands and bogs (Rey et al. 2007).

The diversity of reptiles is surprisingly high for a country with a mostly temperate and continental climate. There are 23 reptile species in Romania, out of which 12 species reach the limit of their geographic range (i.e., *Testudo hermanni*, *Testudo graeca*, *Ablepharus kitaibelii*, *Lacerta trilineata*, *Podarcis tauricus*, *Podarcis muralis*, *Eremias arguta*, *Darevskia praticola*, *Eryx jaculus*, *Dolichophis caspius*, *Elaphe sauromates*, *Vipera ammodytes*), while other two species are near their range edge in Romania (i.e., *Zamenis longissimus*, *Vipera ursinii*) (Gasc et al. 1997).

No updated distribution maps have been published since the publication of the milestone volume on Reptiles in the series Fauna of Romania fifty-two years ago (Fuhn and Vancea 1961), despite a substantial increase in the inventory effort over the following years. However, there are published papers compiling species distributions of single species such as *Darevskia praticola* (Sós et al. 2012), *Vipera ursinii* (Krecsák and Zamfirescu 2008), *Natrix tessellata* (Strugariu et al. 2011), *Emys orbicularis* (Sós 2011), *Testudo hermanni* (Rozylowicz and Dobre 2010), *Eryx jaculus* (Krecsák and Iftime 2006), or restricted to particular geographical areas (e.g., Ghira et al. 2002, Covaciu-Marcov et al. 2006).

We here present an updated overview of the distribution and diversity of reptiles in Romania. The aims of our study are to (1) map the distribution of reptile species in Romania and (2) analyze the spatial pattern of distribution data.

## Materials and methods

### Mapping species occurrences

We extracted the occurrence records from four major sources: published data, museum collections, personal communications from specialists, and our own unpublished field data. The records were primarily stored and managed in a Microsoft Access database, and later imported it in an ESRI file geodatabase using ArcGIS Desktop 10.1 (ESRI, CA). We checked for data quality by (1) filtering the database for doubtful and erroneous records, (2) aggregating the known localities to a finer resolution, and (3) assessing the bias in sampling effort. Our own data were collected over a period of almost 25 years and it involved a large variety of methods. Since the majority of studies carried out were of ecology, the detailed distribution data was not made available in the resulting publications. Our own few publications presenting species distribution data were included in Appendix I. No voucher specimens were collected during our studies.

The distribution records that could not be georeferenced to an actual locality or toponym (e.g., occurrences assigned to mountain ranges, geographical provinces or hydrographic basins) or records with unspecified taxa within genera were not included in the geodatabase. Other doubtful or erroneous records such as species out of their known range or vagrant individuals *sensu* IUCN (2001) were also discarded.

The species taxonomy considered in the present paper is based on Speybroeck et al. (2010). Due to rapid changes in taxonomy, we did not analyze the subspecies in our study, except for *Vipera (Acridophaga) ursinii*, to which detailed studies have confirmed the relevance of taxonomic unit (Ferchaud et al. 2012). While the taxonomic status of *Anguis fragilis* is still under debate, we considered the species complex as a single species (Gvoždík et al. 2010). We encountered a similar problem for *Vipera* (*berus*) *nikolskii*; therefore we did not examine it separately from *Vipera berus* (Zinenko et al. 2010).

We aggregated the occurrence records to the Universal Transverse Mercator (UTM) grid system at a spatial resolution of 25 km^2^ (UTM 5 × 5 km). The records with a spatial resolution of ≤ 25 km^2^ were assigned the corresponding UTM 5 × 5 km grid cell code using primarily the UTM index of localities (Lehrer and Lehrer 1990). The species occurrences with a spatial resolution of > 25 km^2^ were assigned only one grid cell code based on expert knowledge of the species’ habitat requirements (Scott et al. 2002, Franklin 2009) and visual help from the available satellite imagery and an overlaid KMZ file with the UTM 5 × 5 km grid in Google Earth v. 7.0.2 (Google Inc., CA). In order to georeference all records in the geodatabase in ArcGIS Desktop, we created a relationship between the table with species occurrence records and the UTM 5 × 5 km polygon feature class based on the grid cell code as a common attribute.

The occurrences were characterized based on the year of observation into old (if recorded before 1990) and recent (if after 1990, i.e. post Cold-War period) records. The distinction is based on the type of studies published and the style of work. Most papers before 1990 did not indicate the year of observation/collection and some did not even differentiate between new and old records. After 1990 the trend was to publish new locations in pure faunistic studies done within administrative units. Moreover, after 1990 access was not restricted anymore in areas close to the border and it involved also major changes in landscape use and an overall reduction of threats, mostly caused by a reduction in the use of pesticides and fertilizers in agriculture, land abandonment, changes in local hydrology caused by the decline in irrigation intensity and the decline of mining and industrial activities. If the year of observation was not mentioned in the publications, mostly in our national and local journals, then we subtracted 3 to 5 years from the date of publication, based on our estimate of the delay between actual fieldwork and time of publication.

### Spatial patterns in species occurrences

Based on the number of reptile records per UTM 5 × 5 km grid cell, we tested for spatial autocorrelation using Global Moran’s I statistic under the null hypothesis that the occurrence records were evenly distributed. If the null hypothesis is rejected, the occurrence records are more spatially clustered (Z > 0) or dispersed (Z < 0) than expected (Fortin and Dale 2005). We assessed the local patterns of sampling bias using Getis Ord Gi* spatial statistic (Ord and Getis 1995). The metric allowed us to identify the clusters of UTM 5 × 5 cells where the sampling effort was significantly lower (GiZScore < -1.96; i.e., coldspots of occurrences) or higher (GiZScore > 1.96; i.e., hotspots of occurrences) than expected by chance. We set up the distance threshold to 7100 m in order to include the surrounding eight UTM grid cells (Getis and Ord 1992).

In order to assess the altitudinal distribution of each species, we extracted the mean altitude per grid cell from the SRTM digital topographic database (Jarvis et al. 2008) using ArcGIS Desktop Zonal Statistics geoprocessing tool. The grid cells intersecting the Romanian border were excluded from the analysis.

We calculated the species richness at a spatial resolution of 50 × 50 km and 10 × 10 km without considering subspecies separately. Mapping the species richness at a coarser resolution reduced the potential bias in sampling effort and allowed us a better understanding and visualization of regional patterns (Graham and Hijmans 2006).

We then calculated the Extent of Occurrence (EOO) as the minimum convex polygon, and the Area of Occupancy (AOO) as the total area of UTM 5 × 5 km grid cells where a species was reported (IUCN 2001). The exact area of UTM grid cells situated along the border and only partially located in Romania was taken into account in order to avoid overestimation.

Finally, we calculated a rarity index at a 50 × 50 km resolution (RI: Romano et al. 2012) as a measure of species range size in relation to the country area. The rarity index took values between 0 for widespread species and close to 100 for species with a restricted distribution.

All spatial statistics analyses were performed using geoprocessing tools under ArcGIS for Desktop 10.1 (ESRI, CA). Non-spatial statistical analyses were performed in Minitab 16 (Minitab Inc., PA).

## Results

### Species occurrences and ranges

**Figure 1.**
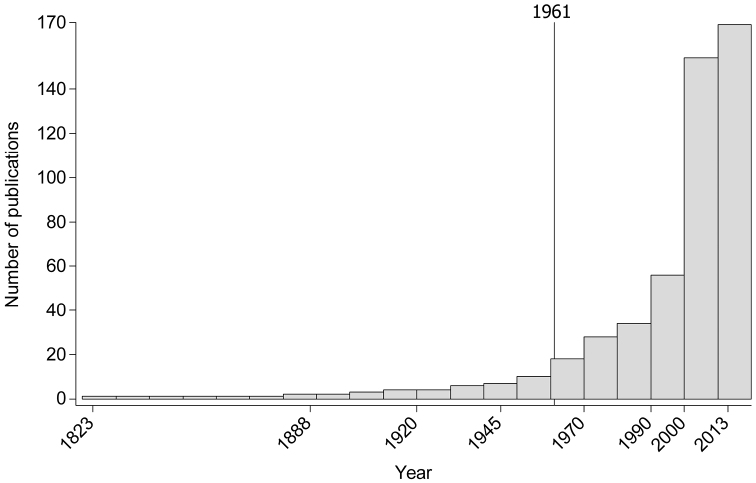
Number of publications with distribution data of reptiles in Romania (1823–2013). The reference line indicates the last country wide assessment published in 1961 (Fuhn and Vancea 1961).

We extracted and georeferenced 18036 reptile records from published papers (66%), museum collections (2%), personal communication from specialists, and our own field surveys (32%). The published papers with the reptile distribution records used in this study are listed in Appendix I, while the dynamics in time of their publication is presented in Figure 1. Among the records, 87.1% were dated after 1990, and 12.9% before 1990 (Appendix II and Table 1). Compared to the national density of reptile records per 100 km^2^ of 0.029 in the GBIF dataset (http://data.gbif.org, accessed 30.03.2013), our database increased it to 7.5 reptile records per 100 km^2^.

The species’ Extent of Occurrence ranged from 6439 km^2^ (*Testudo hermanni*) to 233497 km^2^ (*Natrix natrix*), while species’ Area of Occupancy ranged from 125 km^2^ (*Eryx jaculus*) to 37346 km^2^ (*Lacerta agilis*, Table 1).

**Table 1.** The occurrence records for reptile species in Romania. EOO was estimated as 100% minimum convex polygon, and AOO was estimated as the total area of all UTM grid cell containing species records. Since not all UTM cells matched the 25 km^2^ area, the computed AOO is not a multiple of this value. Rarity index was calculated relative to a 50 × 50 km cell in order to check for regional patterns.

**Species**	**Total number of records**	**Old records (before 1990)**	**New records (after 1990)**	**Number of UTM5 squares**	**EOO (km^2^)**	**AOO (km^2^)**	**Rarity index**
*Emys orbicularis*	753	131	622	561	232748	13491	22.7
*Testudo graeca*	1159	117	1042	154	14441	3748	91.1
*Testudo hermanni*	891	83	808	96	6439	2120	93.4
*Anguis fragilis*	1006	111	895	728	211520	17994	38.2
*Eremias arguta*	85	23	62	30	10851	625	95.1
*Lacerta agilis*	2554	268	2286	1521	231768	37346	13.8
*Darevskia praticola*	93	34	59	60	80161	1449	84.5
*Lacerta trilineata*	133	26	107	64	14028	1589	91.1
*Lacerta viridis*	2737	214	2523	1101	228111	26886	19.5
*Zootoca vivipara*	805	105	700	463	114513	11488	60.9
*Podarcis muralis*	672	111	561	374	167436	9093	56.9
*Podarcis tauricus*	1399	188	1211	358	163668	8671	70.7
*Ablepharus kitaibelii*	250	56	194	100	63728	2413	80.4
*Eryx jaculus*	5	4	1	5	10692	125	96.7
*Coronella austriaca*	527	98	429	389	213819	9581	39.8
*Zamenis longissimus*	500	73	427	363	201245	8868	50.4
*Elaphe sauromates*	47	15	32	27	17099	667	91.8
*Dolichophis caspius*	351	50	301	202	83585	4810	79.6
*Natrix natrix*	2180	163	2017	1361	233497	33202	15.4
*Natrix tessellata*	843	139	704	406	193909	9758	47.9
*Vipera ammodytes*	305	95	210	142	94177	3423	82.9
*Vipera berus*	663	138	525	464	144079	11515	47.1
*Vipera ursinii*	78	35	43	33	67560	729	91.1
**Total**	**18036**	**2277**	**15759**	-	-	-	

### Spatial patterns in species occurrences

**Figure 2.**
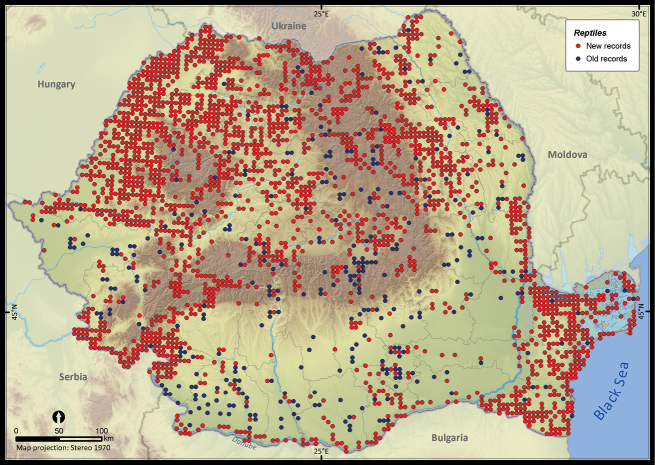
The presence records of reptiles per UTM 5 × 5 km grid cell in Romania. Records reported before 1990 were plotted as old records whereas those reported after 1990 were considered new records.

**Figure 3.**
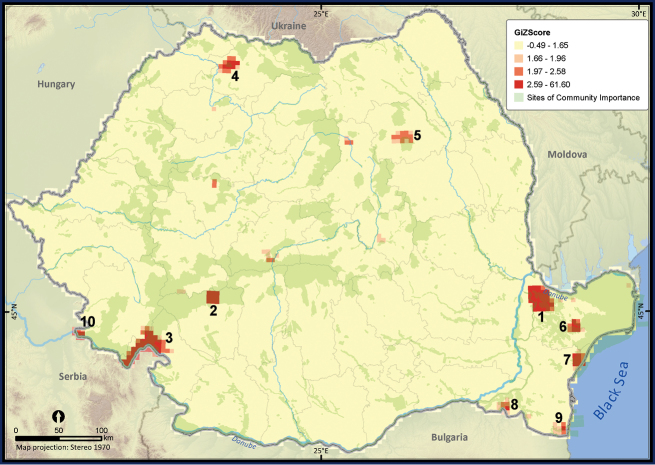
The hotspots of sampling efforts within Romania. The *p* value was < 0.05 when Z scores took values between 1.96 and 61.60, suggesting a highly clustered pattern in the number of reptile occurrences per UTM 5 × 5 km grid cell. The numbered hotspots were: **1** Măcin Mountains **2** Jiului Gorges **3** Iron Gates and Mehedinţi Plateau **4** Sweet chestnut Arboretum of Baia Mare and the surroundings of Baia Mare town **5** Goşman Mountains and the surroundings of Piatra Neamţ town **6** the Danube Delta and Babadag Forest **7** the Danube Delta and Histria Archaeological Complex **8** Canaraua Fetii – Iortmac **9** Hagieni – Cotu Văii Forest **10** Nera river mouth and Baziaş.

Only 27.7% of the total UTM 5 × 5 km grid cells in Romania contained the sighting of at least one reptile species (Figure 2). The number of cumulative reptile records per cell had a clustered pattern, supporting the hypothesis of an overall bias in sampling effort (*Z* = 19.98, *p* < 0.001). Getis Ord Gi* spatial statistic identified several isolated sites, mainly protected areas, as hotspots of sampling effort (Figure 3).

**Figure 4.**
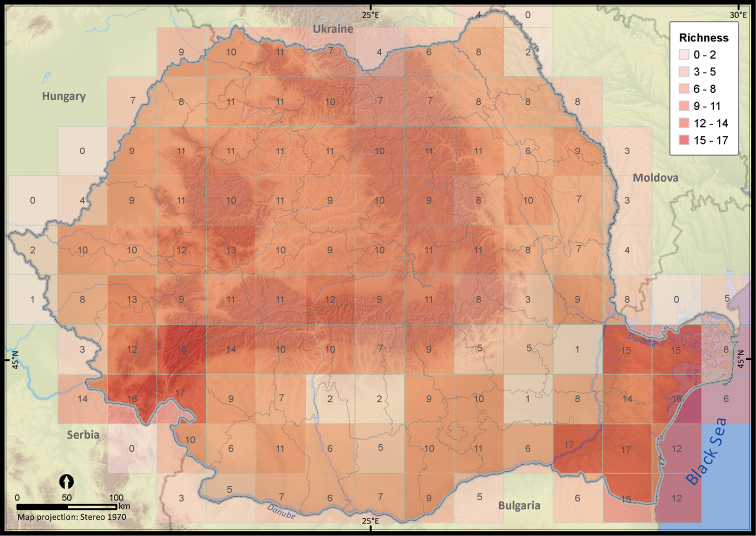
The reptile species richness at a 50 × 50 km grid resolution within Romania.

The species richness map highlighted a lower sampling effort in the vast agricultural areas in the southern part of Romania (Figure 4 and Appendix III). By contrast, the southeastern and southwestern part of Romania, with Mediterranean influences (Rey et al. 2007), presented the highest diversity of reptile species with a maximum richness of 17 species per 50 × 50 km grid cell.

The altitudinal range of reptiles varied between 0 and 2075 m (Figure 5), with only *Zootoca vivipara* and *Vipera berus* occurring above 2000 m, while *Eremias arguta* (occurring at maximum 34 m) and *Eryx jaculus* (occurring at maximum 88 m) are clearly restricted to lowlands. The updated distribution maps of reptile species in Romania are presented in Figures 6–28.

**Figure 5.**
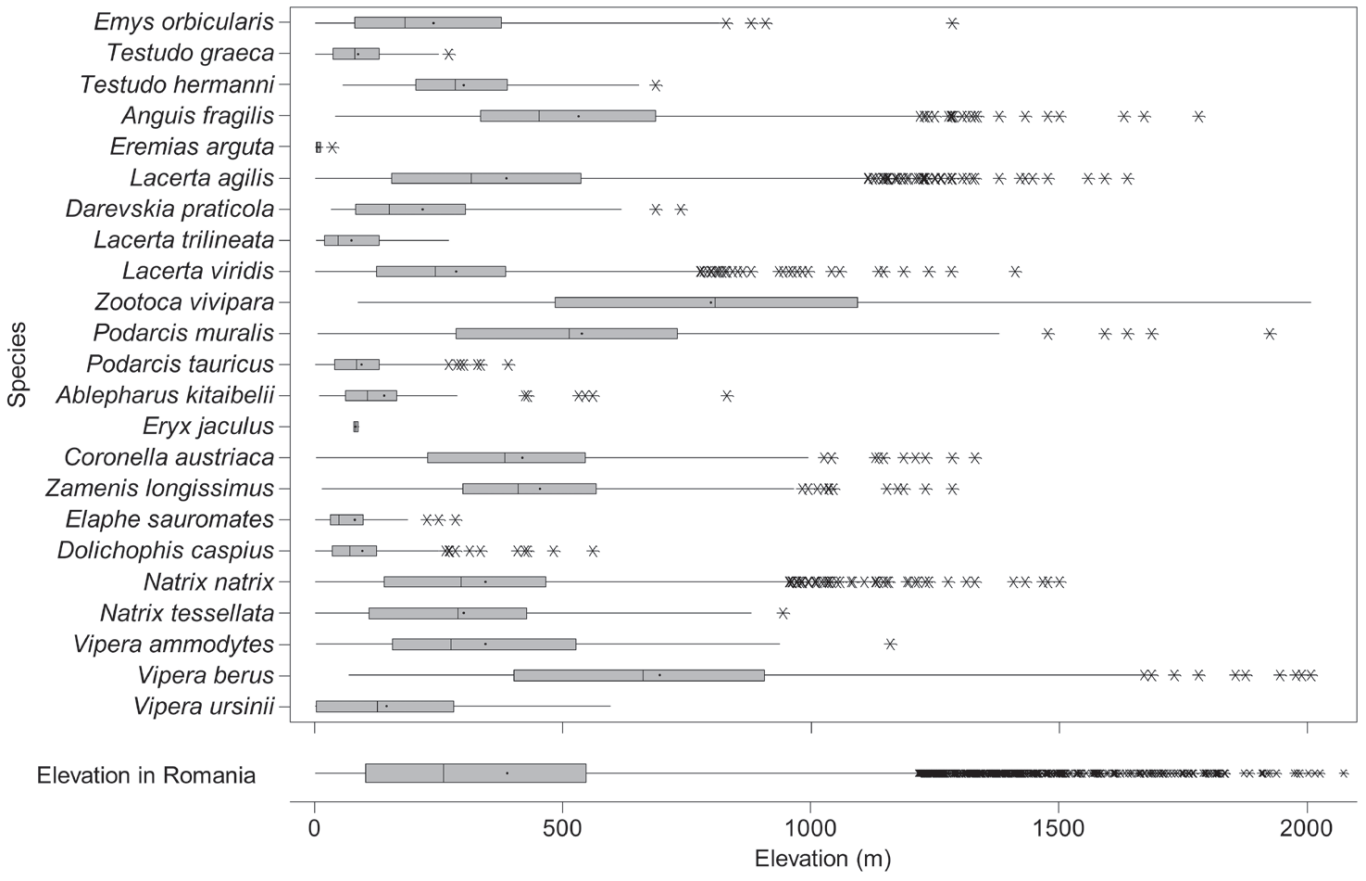
The box and whisker plot of altitudinal distribution of reptile species in Romania. The boxes represent 25^th^–75^th^ percentiles, upper and lower whiskers extends minimum and maximum data point within 1.5 box heights from the bottom and from the top of the boxes. Asterisks indicate outliers, lines and dots inside the boxes denote medians and means, respectively.

**Figure 6.**
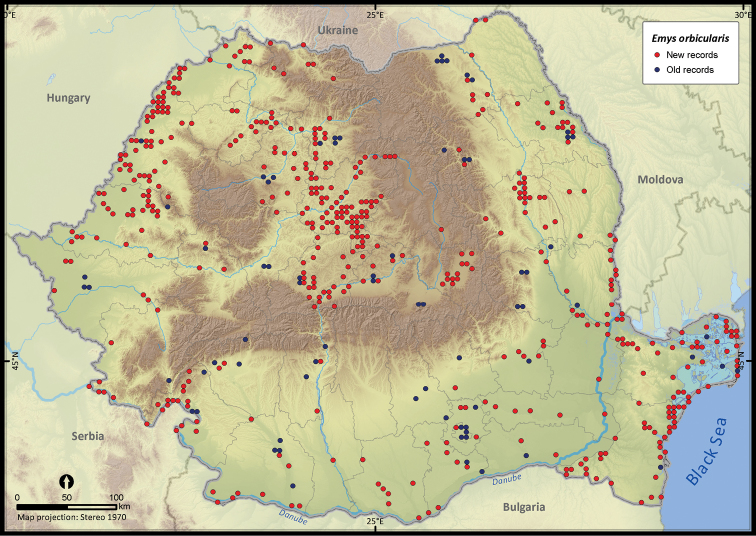
*Emys orbicularis*.

**Figure 7.**
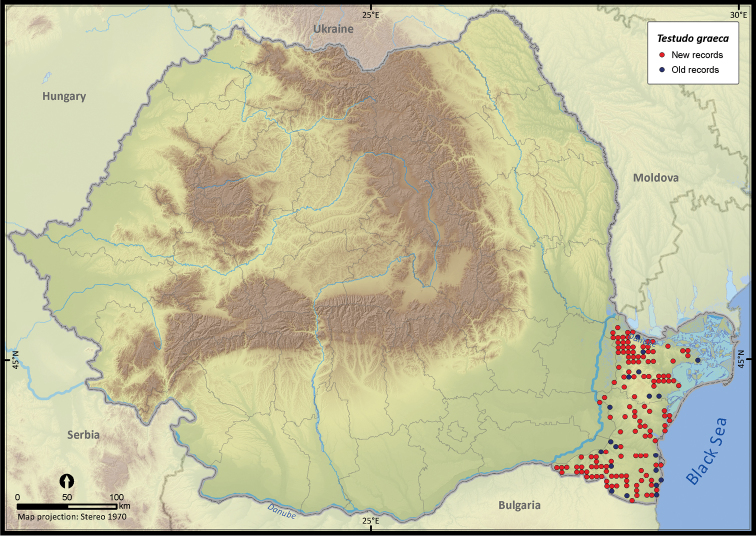
*Testudo graeca*.

**Figure 8.**
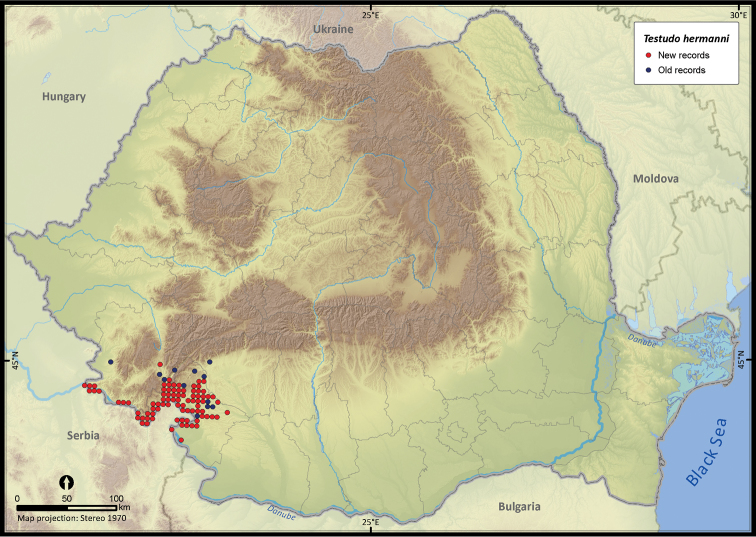
*Testudo hermanni*.

**Figure 9.**
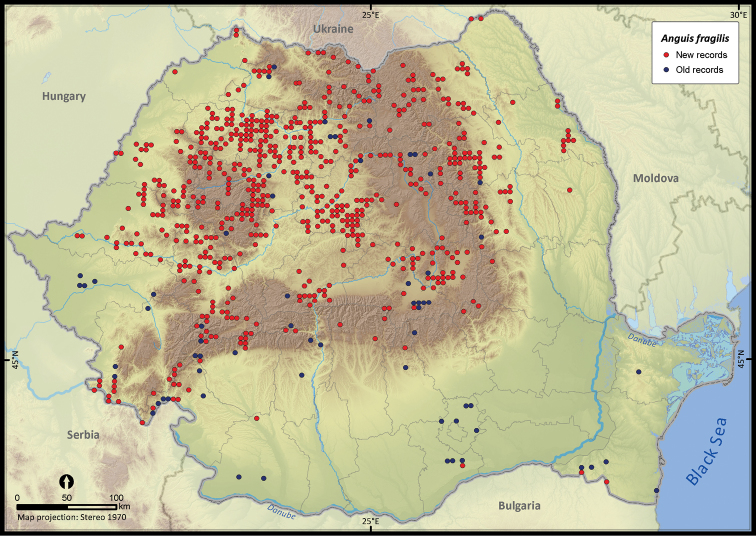
*Anguis fragilis*.

**Figure 10.**
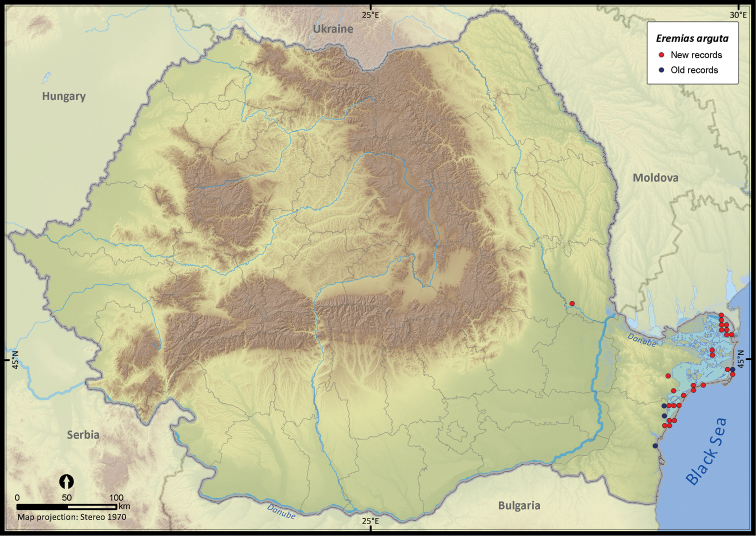
*Eremias arguta*.

**Figure 11.**
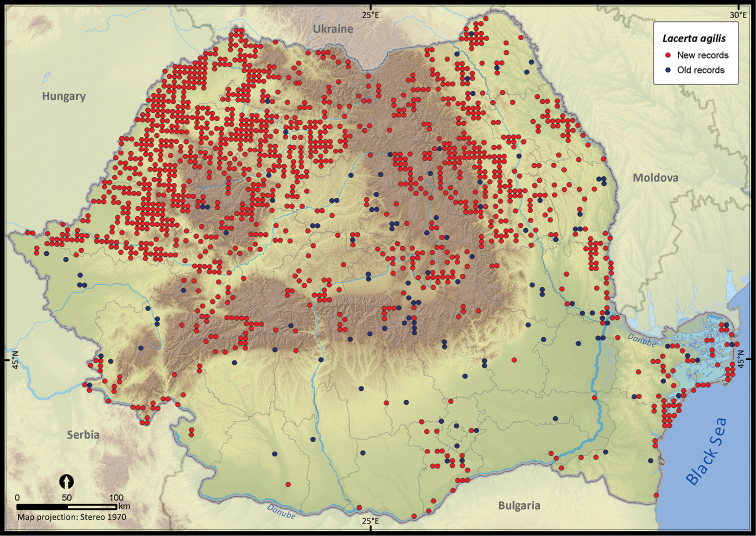
*Lacerta agilis*.

**Figure 12.**
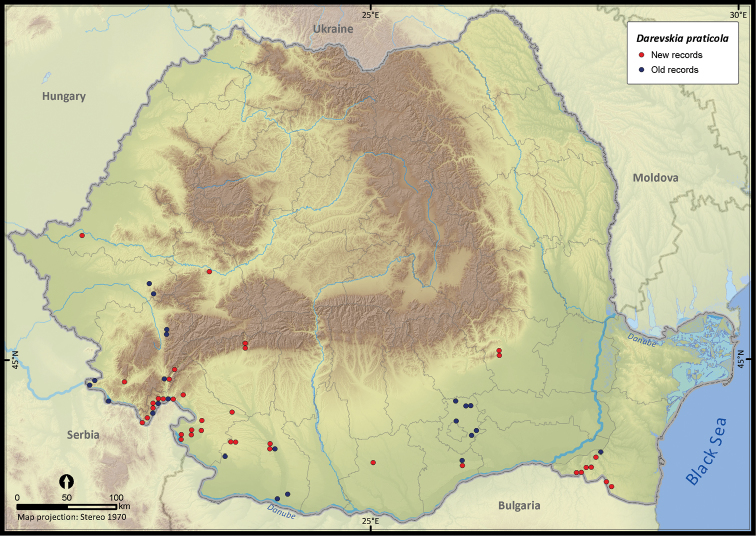
*Darevskia praticola*.

**Figure 13.**
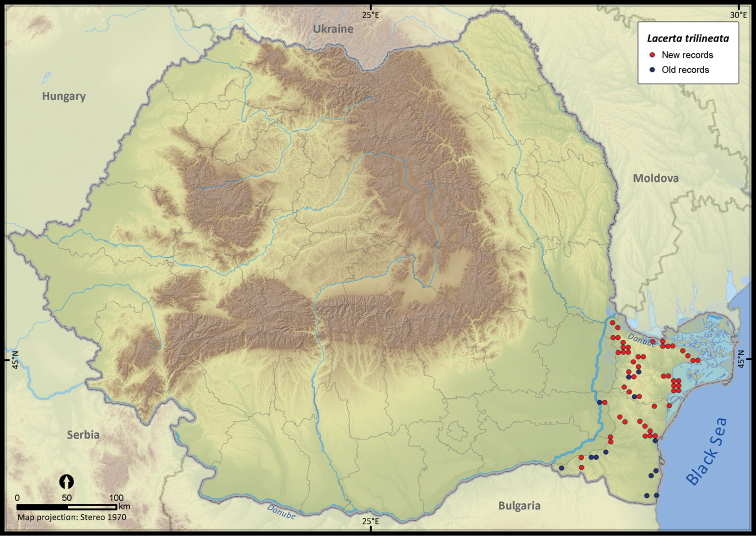
*Lacerta trilineata*.

**Figure 14.**
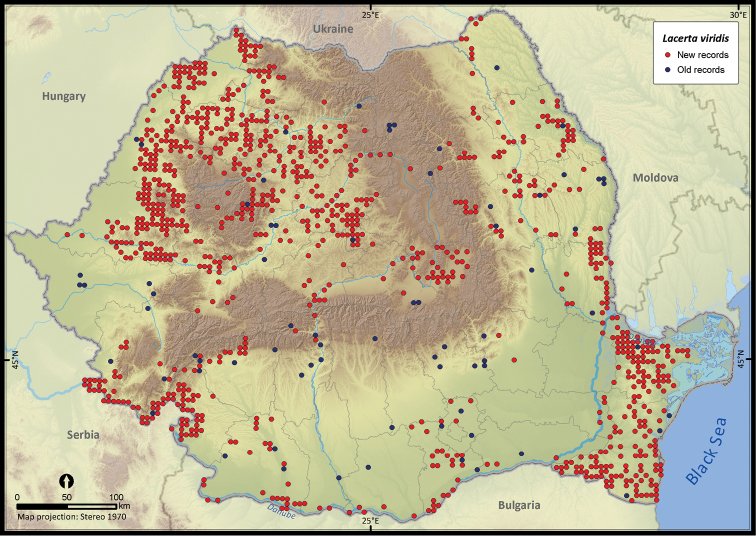
*Lacerta viridis*.

**Figure 15.**
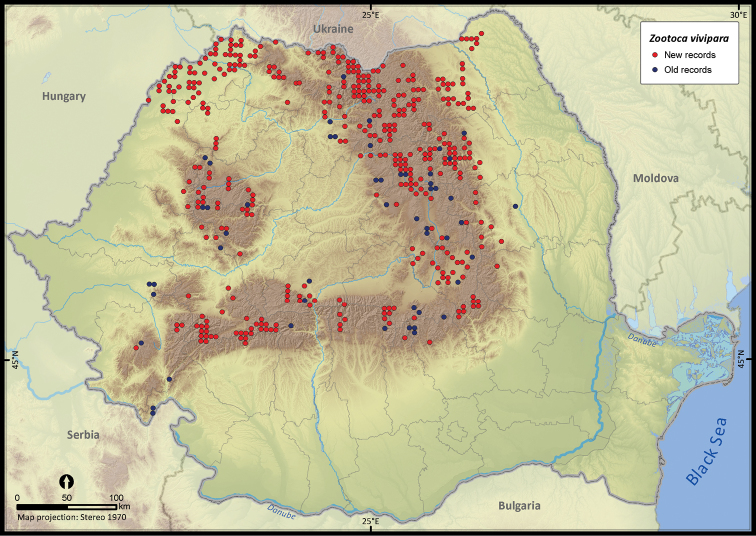
*Zootoca vivipara*.

**Figure 16.**
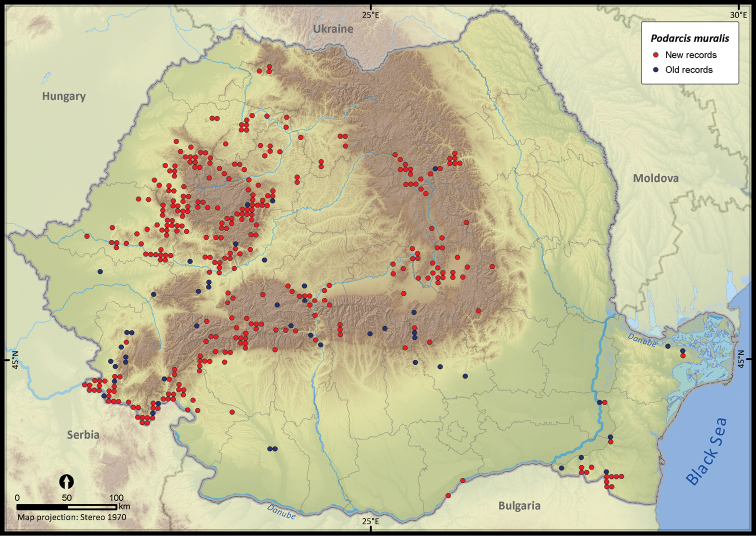
*Podarcis muralis*.

**Figure 17.**
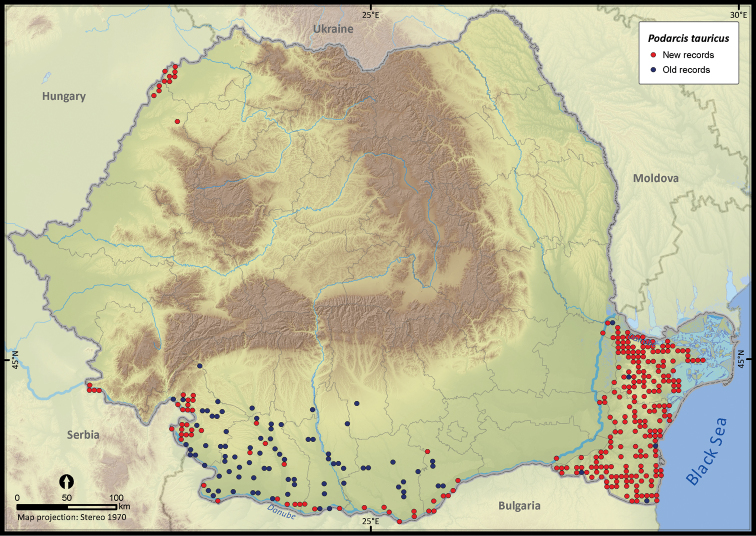
*Podarcis tauricus*.

**Figure 18.**
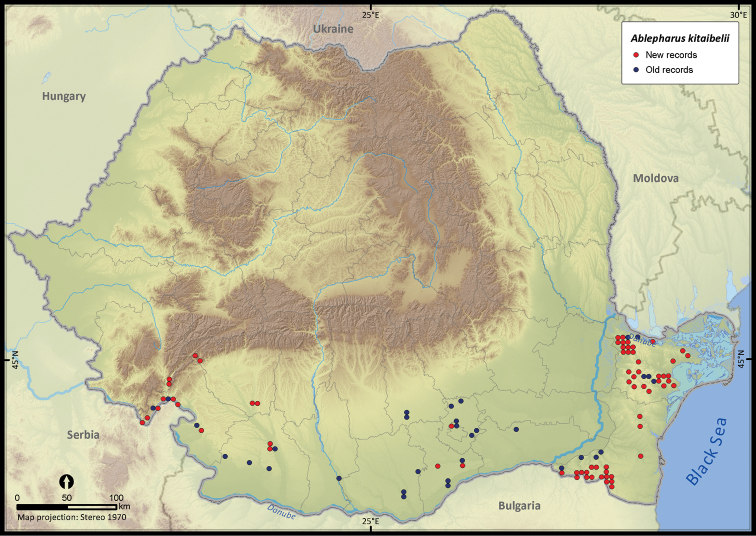
*Ablepharus kitaibelii*.

**Figure 19.**
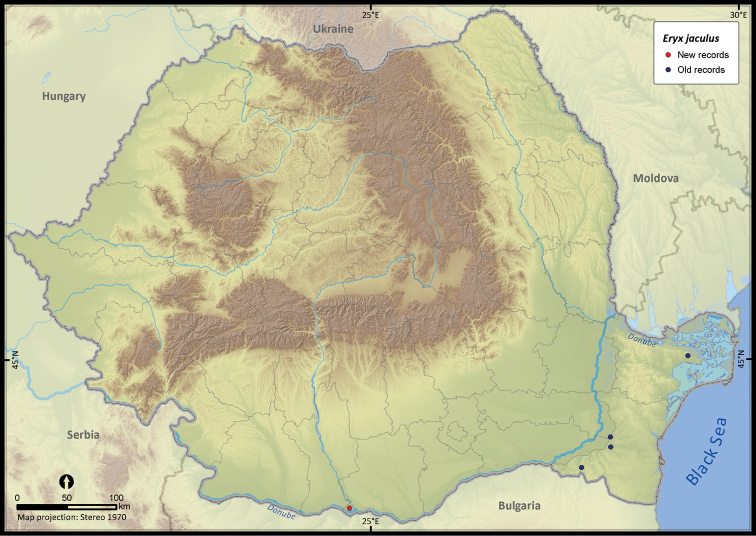
*Eryx jaculus*.

**Figure 20.**
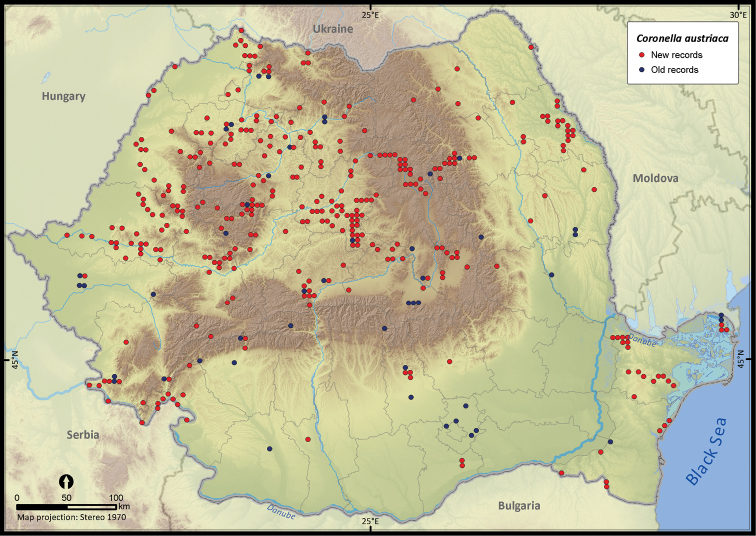
*Coronella austriaca*.

**Figure 21.**
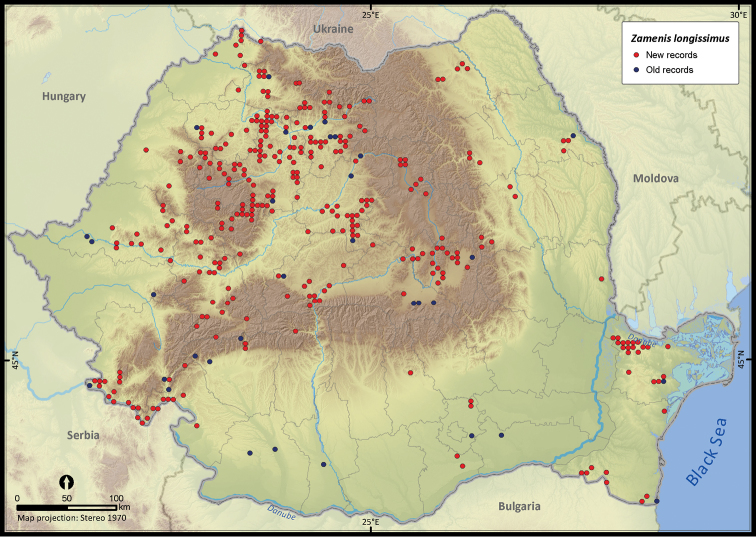
*Zamenis longissimus*.

**Figure 22.**
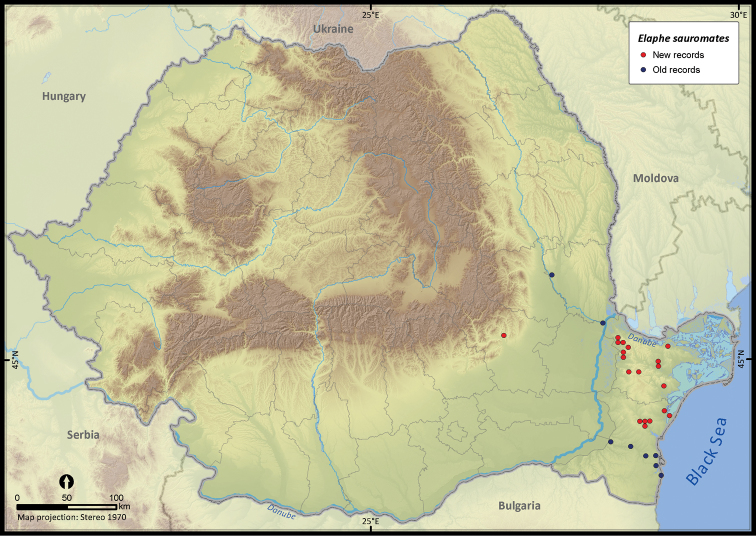
*Elaphe sauromates*.

**Figure 23.**
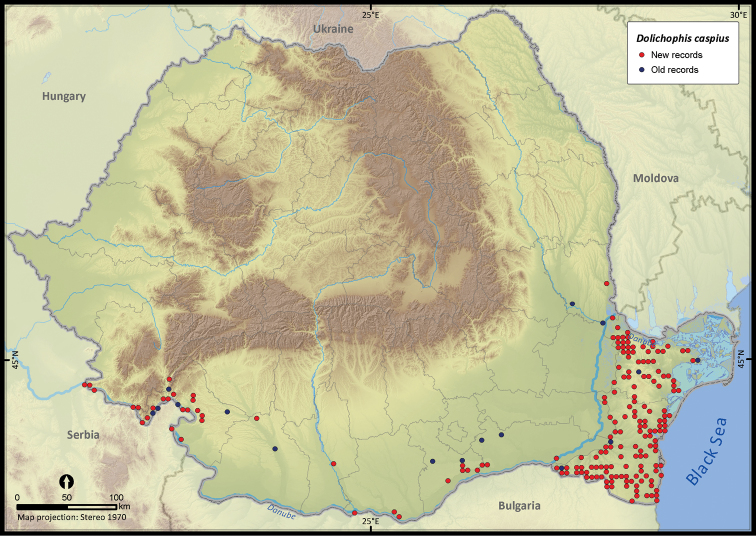
*Dolichophis caspius*.

**Figure 24.**
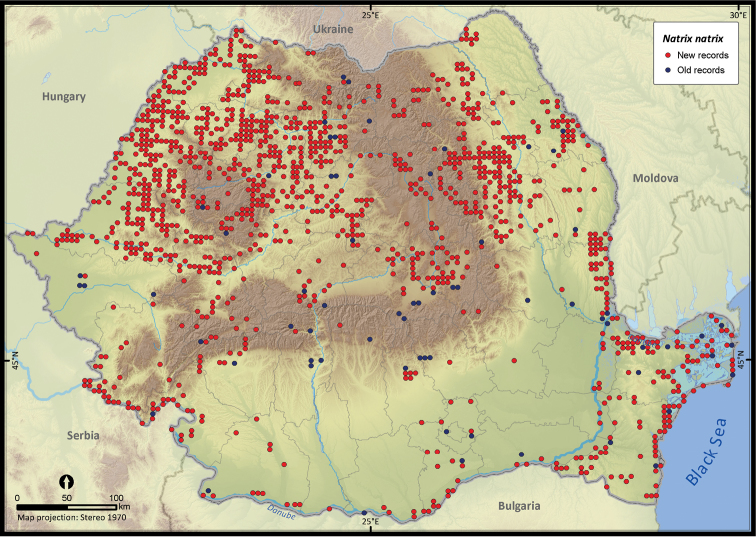
*Natrix natrix*.

**Figure 25.**
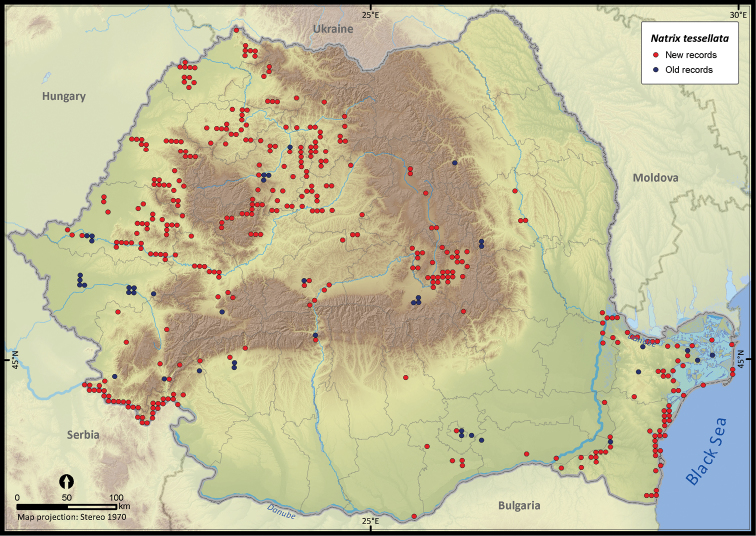
*Natrix tessellata*.

**Figure 26.**
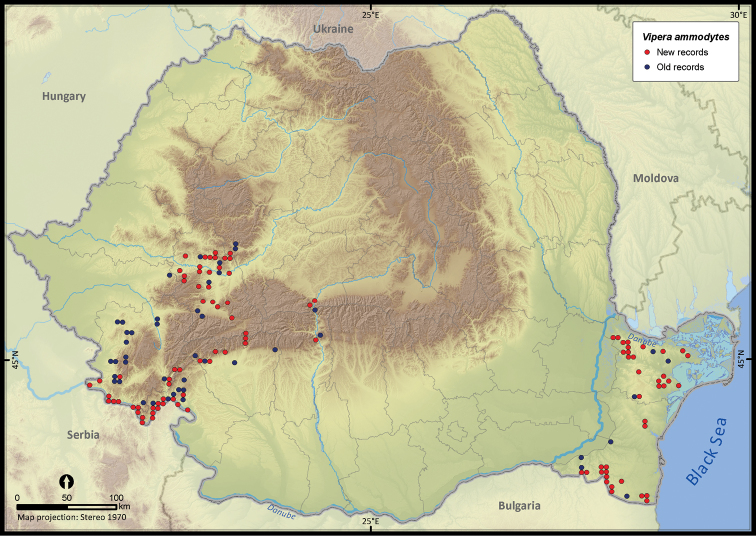
*Vipera ammodytes*.

**Figure 27.**
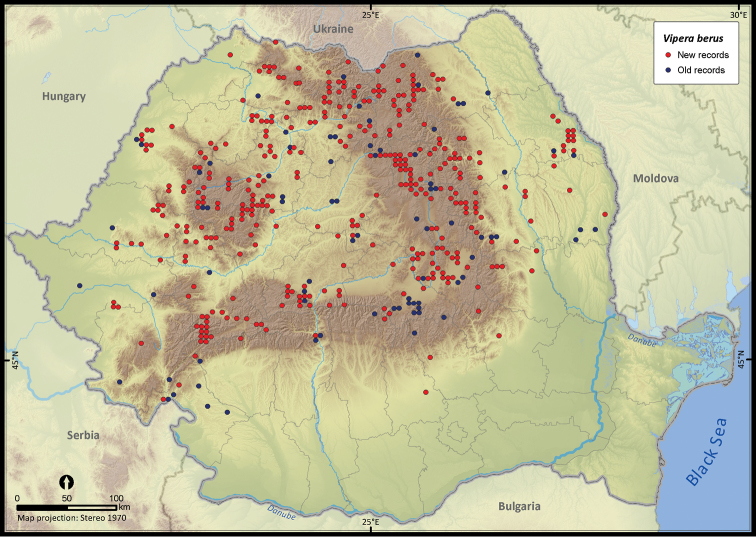
*Vipera berus*.

**Figure 28.**
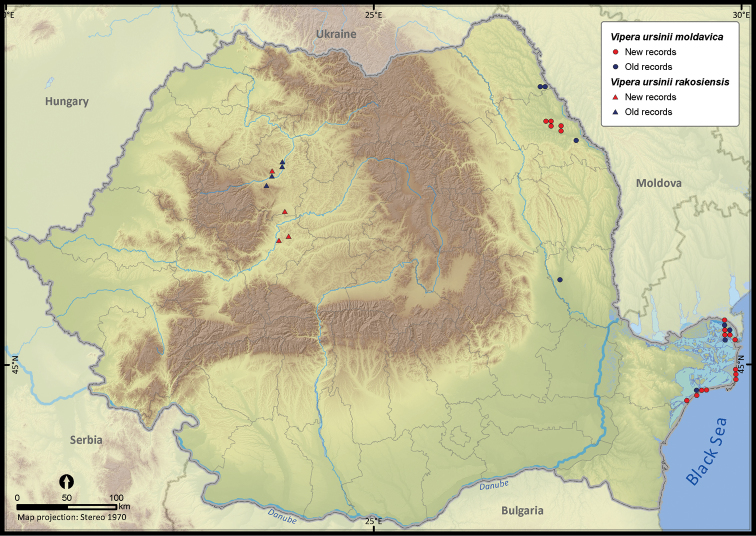
*Vipera ursinii moldavica* and *Vipera ursinii rakosiensis*.

## Discussion

We here present the first comprehensive distribution database of the reptile species occurring in Romania. We fulfilled the major requirements of data quality by (1) filtering the large amount of data for doubtful and erroneous records, (2) aggregating the known localities to a fine resolution of 25 km^2^, and (3) assessing the bias in sampling effort and thus providing useful information for further analyses.

We addressed a series of issues related to the quality and relevance of occurrence records. The most conspicuous issue was the overall biased density of records, which might appear due to differences in species detectability. For instance, our database showed that the snake species with low detectability (Hartel et al. 2009, Durso et al. 2011) had a low number of occurrences despite their wide range. Notable exceptions are the grass snake and the dice snake (genus *Natrix*), both of them being active during the day and easy to identify. The higher detectability of most lizards and both tortoise species resulted in a large number of occurrences and thus, more detailed distribution maps.

Another issue of concern was misidentification (e.g., Török 2012), due to difficulties in distinguishing the actual species based only on sightings. For example, it is rather difficult to distinguish among green lizards, especially between *Lacerta viridis* and *Lacerta trilineata* and we assumed that some of the occurrence records for those species were misallocated.

Although individuals of *Testudo hermanni* were reported from south-east Romania (Iftime 2002, Sós et al. 2008), we chose not to map the respective occurrences, considering them a case of vagrancy (Rozylowicz and Dobre 2010). The presence report of *Testudo hermanni* from Hunedoara county (Togănel 1993) was also not considered since it was the case of a recent human introduction into the wild (Rozylowicz 2008).

The analysis of sampling effort revealed a significantly higher number of sightings per grid cell within certain areas, mostly protected areas, such as the Jiului Gorges National Park, the Iron Gates Natural Park or the Măcin Mountains National Park. That was a common pattern previously reported from other countries and for different taxa (e.g., Loureiro et al. 2010, Botts et al. 2011). Despite those hotspots, the sampling effort was balanced across the country; therefore the dataset might be useful for additional analyses with only a simple trimming procedure required (Peterson et al. 2011). In the case of amphibians (Cogălniceanu et al. 2013), the sampling effort was biased towards the same hotspots but their number was significantly less numerous for reptiles. Nevertheless, the patterns in species richness support a similar statement revealing a higher richness in the warmer and drier climate in the south-west and the south-east parts of Romania (e.g., Rodríguez et al. 2005) and a constant richness in the rest of the country. Two gaps that require further investigations were revealed in the southern part of Romania (Oltenia and Bărăgan plains), probably being determined by the lack of research interest in those regions, due to their dominant agricultural landscape (Rey et al. 2007, Iojă et al. 2011). The gaps in reptile richness were similar to those of amphibian richness (Cogălniceanu et al. 2013).

The number of reptile species observed in Romania might increase in the near future with two species, namely *Pseudopus apodus* (Lepşi 1926, Cogălniceanu et al. 2008) and *Mediodactylus kotschyi*, bothpresent in Bulgaria and near the Romanian border (Gasc et al. 1997).

Several species were widespread across the country (e.g., *Emys orbicularis*, *Lacerta viridis*, *Lacerta agilis*, *Natrix natrix*, *Natrix tessellata*, *Zamenis longissimus*, *Vipera berus*), while others occurred only in the south of Romania (e.g., *Testudo hermanni*, *Testudo graeca*, *Eremias arguta*, *Lacerta trilineata*, *Eryx jaculus*, *Elaphe sauromates*, *Dolichophis caspius*). *Lacerta viridis* had the largest number of sightings mainly because it is a highly detectable species. Its AOO was surpassed only by *Lacerta agilis* and *Natrix natrix*, two other highly detectable species. *Eryx jaculus* is the rarest reptile in the country (see Table 1) with a single new report after 1990, based on a road-kill specimen (Covaciu-Marcov et al. 2012).

The different biogeographic regions overlapping in Romania will face different types and levels of these changes (Popescu et al. in press). Climate change may cause a shift in reptile ranges, although the Carpathian Mountains and the Steppic and Black Sea province of Dobrogea will act as a refuge, being considered critical areas for conservation (Araújo et al. 2006, Popescu et al. in press). To alleviate these threats by conservation activities (e.g., by establishing new protected areas), a key step is predicting the range responses to different climate change scenarios (Franklin 2009, Araújo et al. 2011). Our dataset provides a robust starting point for such analyses. Climate change alone does not result in a rapid range shift; instead, additionally, habitat loss due to the abandonment of traditional agricultural activities such as manual mowing, low-intensity grazing, small-size orchards and vineyards (Plieninger et al. 2006, Angelstam et al. 2013) might lead to a rapid contraction of the reptiles ranges (e.g., *Testudo hermanni*, Rozylowicz and Popescu 2013). Thus, our database also allows for further investigations resulting in concrete conservation activities.

## Appendix I

The publications used to compile the distribution of reptile species native to Romania. (doi: 10.3897/zookeys.341.5502.app1) File format: Microsoft Word file (doc).

## Appendix II

The reptiles occurrence records within Romania per time intervals (1961 represented the publishing year of the last country wide assessment). (doi: 10.3897/zookeys.341.5502.app2) File format: Microsoft Word file (doc).

## Appendix III

The reptile species richness at a 10 × 10 km grid resolution within Romania (SCI = Natura 2000 Sites of Community Importance). (doi: 10.3897/zookeys.341.5502.app3) File format: Microsoft Word file (doc).
