# Supplementary material for: Diversity and distribution of reptiles in Romania
Source: Zookeys. 2013 Oct 8;(341):49–76. doi: 10.3897/zookeys.341.5502 (PMC3800809; doi:10.3897/zookeys.341.5502)
Supplement: Supplementary file 1 — The publications used to compile the distribution of reptile species native to Romania. (doi: 10.3897/zookeys.341.5502.app1) File format: Microsoft Word file (doc). [file ZooKeys-341-049-s001.doc]

**Appendix 1**

**The publications used to compile the distribution of reptile species native to Romania.**

Andrei M (1997) Note on the herpetofauna of the Maramureş (Romania). Travaux du Muséum National d'Histoire Naturelle "Grigore Antipa" 37: 129-133.

Andrei M (2002) Contributions to the knowledge of the herpetofauna of southern Dobruja (Romania). Travaux du Muséum National d'Histoire Naturelle "Grigore Antipa" 44: 357-373.

Ardelean G, Trifonov P (2002) Vertebratele din Ţara Făgăraşului. Satu-Mare, Studii şi Comunicări, Seria Ştiinţele Naturii 2-3: 197-204.

Băcescu M (1934) Contributions a la faune des reptiles de Dobrodgea. Annales Scientifiques Universite Jassy 19: 317-330.

Băcescu M (1959) La faune relique des sources se trouvant a l'extremite ouest du Tekirghiol et le probleme s'une reservation naturelle dans cet endroit. În Lucrările Sesiunii Ştiinţifice a Staţiunii Zoologice Marine "Prof I Borcea"-Agigea, 15-17 sept 1956. Iaşi, 1-7 pp.

Băcescu M, Matei D (1958) Şarpele de apă (*Natrix tessellata* L) în bazinul superior al Bistriţei moldoveneşti. Natura 10: 137-139.

Bănăduc D (2006) Colecţia de reptile a Muzeului de Istorie Naturală Sibiu. Brukenthal Acta Musei 1: 175-179.

Béres I (1997) Contribuţii la cunoaşterea faunei amfibienilor şi reptilelor din Depresiunea Maramureşului şi protecţia lor. Nymphaea Folia naturae Bihariae 23-25: 151-154.

Bielz EA (1888) Die Fauna der Wirbeltiere Siebenbürgens nach ihrem jetzigen Bestande. Verhandlungen und Mitteilungen des Siebenbürgischen Vereins für Naturwissenschaften zu Hermannstadt 38: 15-120.

Borcea M (1983) Fauna de amfibii şi reptile din Munţii Rodnei. Prezentare zoogeografică. În Pascu S, Negruţiu E (Eds) Rezervaţia Pietrosul Rodnei la 50 ani. Academia Repubicii Socialiste România. Filiala Cluj-Napoca; Comitetul de Cultură şi Educaţia Socialistă Maramureş, Baia-Mare, 120-127.

Călinescu RI (1931) Contribuţiuni sistematice şi zoogeografice la studiul amphibiilor şi reptilelor din România. Academia Română Memoriile Secţiunii Ştiinţifice, Seria III 7: 119 -291.

Cicort-Lucaciu AS, Covaciu-Marcov SD, Bogdan HV, Sas I (2012) Implication upon herpetofauna of a road and its reconstruction in Carei Plain Natural Protected Area (Romania). Ecologia Balkanica 4: 99-105.

Cîrlig T (1988) Extinderea arealului Viperidelor în judeţul Arad. Ziridava 17: 161-162.

Cogălniceanu D, Ghira I, Ardeleanu A (2001) Spatial distribution of herpetofauna in the Retezat Mountains National Park–Romania. Biota 2: 9-16.

Cogălniceanu D, Băncilă R, Samoilă C, Hartel T (2008) The current distribution of herpetofauna in the Maramureş County and the Maramureş Mountains Nature Park, (Maramureş, Romania). Transylvanian Review of Systematical and Ecological Research. The Maramureş Mountains Nature Park 5: 189-200.

Cogălniceanu D, Băncilă R, Samoilă C, Hartel T, Tudor M (2007) The herpetofauna of the Maramureş Mountains Natural Park. ENSFI Conference Proceedings. Baia Mare, Romania. pp. 300-305.

Covaciu-Marcov SD (1999) Contribuţii la studiul herpetofaunei dealului Şomleu. Analele Universităţii din Oradea, Fascicula Biologie 6: 175-190.

Covaciu-Marcov SD (2002) Date preliminare privind herpetofauna Văii Ierului. Satu-Mare, Studii şi Comunicări, Seria Ştiinţele Naturii II-III: 191-196.

Covaciu-Marcov SD (2010) Data upon the presence of *Ablepharus kitaibelli* in the Getic Piedmont, Gorj County. North-Western Journal of Zoology 6: 316-318.

Covaciu-Marcov SD, Bogdan HD, Paina C, Toader S, Condure N (2008) The herpetofauna of the north-western region of Bihor County, Romania. Biharean Biologist 2: 5-13.

Covaciu-Marcov SD, Cicort-Lucaciu AS, Ferenti S, David A (2008) The distribution of lowland *Zootoca vivipara* populations in North-Western Romania. North-Western Journal of Zoology 4: 72-78.

Covaciu-Marcov SD, Cicort-Lucaciu AS, Gaceu O, Sas I, Ferenti S, Bogdan HV (2009) The herpetofauna of the south-western part of Mehedinţi County, Romania. North-Western Journal of Zoology 5: 142-164.

Covaciu-Marcov SD, Cicort-Lucaciu AS, Ile RD, Paşcondea A, Vatamaniuc R (2007) Contributions to the study of the geographical distribution of the herpetofauna in the North-East area of Arad County, Romania. Herpetologica Romanica 1: 62-69.

Covaciu-Marcov SD, Cicort-Lucaciu AS, Lazăr V, Szeibel N, Balaj L (2007) The herpetofauna of the lower hydrographical basin of Crişul Alb, the district of Arad (Romania). Oltenia, Studii şi Comunicări, Ştiinţele Naturii 23: 143-147.

Covaciu-Marcov SD, Cicort-Lucaciu AS, Sas I, Bogdan HV, Pusta C (2003) Preliminary data about the distribution of *Podarcis taurica* in the North-Western parts of Romania. Analele Universităţii din Oradea, Fascicula Biologie 10: 111-117.

Covaciu-Marcov SD, Cicort-Lucaciu AS, Sas I, Bredet AM, Bogdan HV (2005) Herpetofauna from the basin of Mureş river in Arad county, Romania. Environment & Progress 5: 147-152.

Covaciu-Marcov SD, Cicort-Lucaciu AS, Sas I, Groza MI, Bordaş I (2007) Contributions to the knowledge regarding the herpetofauna from the Maramureş county areas of ”Măgura Codrului”, Romania. Biharean Biologist 1: 50-56.

Covaciu-Marcov SD, Cicort-Lucaciu AS, Sas I, Moşu AG, Toth B (2008) Contributions to the knowledge of the composition and geographical distribution of the Western Maramureş County Herpetofauna. Herpetologica Romanica 2: 27-36.

Covaciu-Marcov SD, Cicort-Lucaciu AS, Sas I, Strugariu A, Cacuci P, Gherghel I (2008) Contributions to the knowledge regarding the composition and geographical distribution of the herpetofauna from Northern Moldavia (Suceava and Botosani Counties, Romania). North-Western Journal of Zoology 4: 25-47.

Covaciu-Marcov SD, David A (2009) *Dolichophis caspius* (Serpentes: Colubridae) in Romania: New distribution records from the northern limit of its range. Turkish Journal of Zoology 33: 1-3.

Covaciu-Marcov SD, Ferenti S, Ghira I, Sas I (2012) *Eryx jaculus* (Reptilia, Boidae) north of Danube: a road-killed specimen from Romania. Acta Herpetologica 7: 41-47.

Covaciu-Marcov SD, Ferenti S, Ghira I, Sas I (2012) High road mortality of *Dolichophis caspius* in southern Romania. Is this a problem? North-Western Journal of Zoology 8: 370-373.

Covaciu-Marcov SD, Ghira I, Cicort-Lucaciu AS, Sas I, Strugariu A, Bogdan HV (2006) Contributions to knowledge regarding the geographical distribution of the herpetofauna of Dobrudja, Romania. North-Western Journal of Zoology 2: 88-125.

Covaciu-Marcov SD, Ghira I, Sas I (2004) Contribuţii la studiul herpetofaunei zonei Oaşului (judeţul SM, România). Environment & Progress 2: 107-112.

Covaciu-Marcov SD, Ghira I, Venczel M (2000) Contribuţii la studiul herpetofaunei din zona Oradea. Nymphaea Folia naturae Bihariae 28: 143-158.

Covaciu-Marcov SD, Kovacs I, Cicort-Lucaciu A, Sas I, Secare P (2009) Data upon the composition and the geografic distribution of the herpetofauna of the Almaş-Agrij Depression (Salaj county, Romania). Oltenia, Studii şi Comunicări, Ştiinţele Naturii 25: 173-179.

Covaciu-Marcov SD, Sas I, Cicort-Lucaciu A, Bogdan HV, Ardelean R (2006) The herpethofauna of the north-western region of Sălaj county, Romania. Studii şi Cercetări Ştiinţifice Universitatea din Bacău Seria Biologie 11: 85-90.

Covaciu-Marcov SD, Sas I, Cicort-Lucaciu A, Bogdan HV, Groza M (2006) Contribuţii la cunoaşterea compoziţiei şi răspândirii herpetofaunei Moldovei dintre Siret şi Prut. Oltenia, Studii şi Comunicări, Ştiinţele Naturii 22: 242-247.

Covaciu-Marcov SD, Sas I, Cicort-Lucaciu A, Kovacs EH (2003) Notes upon the herpetofauna of the Northern area of the Botoşani county (Romania). Studii şi Cercetări Ştiinţifice Universitatea din Bacău Seria Biologie 8: 201-205.

Covaciu-Marcov SD, Sas I, Cicort-Lucaciu A, Peter I, Bogdan HV (2005) Notes upon the herpetofauna of the south-west area of the county of Caraş-Severin, Romania. Revue Roumaine de Biologie Série de biologie animale 50: 47-56.

Covaciu-Marcov SD, Sas I, Cicort-Lucaciu AS, Kovacs EH, Pintea C (2009) Herpetofauna of the Natural Reserves from Carei Plain: zoogeographical significance, ecology, statute and conservation. Carpathian Journal of Earth and Environmental Sciences 4: 69-80.

Covaciu-Marcov SD, Sas I, Cupşa D, Meleg G, Bud B (2003) Studii herpetologice în regiunea Munţilor Pădurea Craiului şi Plopişului (jud. Bihor, România). Analele Universităţii din Oradea, Fascicula Biologie 10: 81-95.

Covaciu-Marcov SD, Sas I, Kiss A, Bogdan HV, Cicort-Lucaciu AS (2006) The herpetofauna from the Teuz River hydrographic basin (Arad county, Romania). North-Western Journal of Zoology 2: 27-38.

Covaciu-Marcov SD, Sas I, Lazar V, Szeibel N, Condure N (2008) The herpetofauna in the plain area from the western Satu Mare county, Romania. Oltenia, Studii şi Comunicări, Ştiinţele Naturii 24: 161-166.

Covaciu-Marcov SD, Telcean I, Cupşa D, Cadleţ D, Zsurka R (2002) Contribuţii la studiul herpetofaunei din regiunea Marghita (jud. Bihor, România). Analele Universităţii din Oradea, Fascicula Biologie 9: 47-58.

Covaciu-Marcov SD, Telcean I, Cupşa D, Sas I, Cicort-Lucaciu AS (2003) Contribuţii la cunoaşterea herpetofaunei regiunii bazinului hidrografic mediu şi inferior al Crişului Negru (jud. Bihor, România). Oltenia, Studii şi Comunicări, Ştiinţele Naturii 19: 189-194.

Covaciu-Marcov SD, Telcean I, Sala G, Sas I, Cicort-Lucaciu A (2003) Contribuţii la cunoaşterea herpetofaunei regiunii Beiuş, jud. Bihor, România. Nymphaea Folia naturae Bihariae 30: 127-141.

Cruce M (1971) Contribuţii la studiul faunei herpetologice din Oltenia. Analele Universităţii din Craiova, III, Ştiinţe agricole şi biologice 3: 389-393.

Cruce M, Răducanu I (1976) Reproducerea la broasca ţestoasă de uscat (*Testudo hermanni hermanni* G). Studii şi cercetări de biologie Seria Biologie Animală 28: 175-180.

Cuzic M (2004) Contribuţii la studiul mamiferelor şi reptilelor din zona limanelor fluviale Bugeac, Oltina, Dunăreni şi Vederoasa. Delta Dunării-Studii şi cercetări de ştiinţele naturii şi muzeologie 2: 167-174.

Dehelean I, Ardelean G (2000) Herpetofauna zonei Firiza (Baia Mare). Satu-Mare, Studii şi Comunicări, Seria Ştiinţele Naturii I: 155-159.

Falcă M, Vasiliu-Oromulu L, Sanda V, Paucă-Comănescu M, Honciuc V, Maican S, Purice D, Dobre A, Stănescu M, Onete M, Biţă-Nicolae C, Matei B, Codrici I (2004) Ecosystemic characterization of some flooting ash forests from the Neajlov Holm (Giurgiu district). Proceedings of the Institute of Biology 6: 59-71.

Fejérváry-Lángh AM (1943) Beiträge und Berichtigungen zum Reptilien-Teil des ungarischen Faunenkataloges. Fragmentum Fauna Hungariae 6: 81-98.

Frivaldszky E (1823) Monographia Serpentum Hungarye. Typis Nobilis Joannis Thomae Trattner de Petróza, Budapest, 62 pp.

Fuhn IE (1964) Situaţia actuală a faunei noastre de amfibieni şi reptile şi ocrotirea ei. Ocrotirea naturii şi a mediului înconjurător 8: 231-248.

Fuhn IE (1970) Amfibii şi reptile din zona viitorului lac de baraj de la Porţile de Fier. Studii şi cercetări de biologie Seria Zoologie 22: 321-332.

Fuhn IE (1970) Aspecte ale situaţiei actuale a faunei din rezervaţia Pădurea Hagieni. Ocrotirea naturii şi a mediului înconjurător 14: 65-68.

Fuhn IE (1986) Melanism şi anomalii ale folidozei la un exemplar de *Vipera ammodytes ammodytes* (L. 1758) (Viperidae, Serpentes, Reptilia) din împrejurimile oraşului Reşiţa. Studii şi cercetări de biologie Seria Biologie Animală 38: 7-10.

Fuhn IE, Cristurean I (1977) Situaţia actuală a rezervaţiei naturale Pădurea Hagieni. Ocrotirea naturii şi a mediului înconjurător 21: 103-110.

Fuhn IE, Hîrşu M (1962) *Lacerta praticola* pontica Lantz şi Cyren, o şopîrlă nouă pentru fauna herpetologică a Dobrogii. Natura 5: 39-41.

Fuhn IE, Vancea Ş (1961) Reptilia. Fauna RPR, 14(2). Editura Academiei RPR, Bucureşti, 352 pp.

Fuhn IE, Vancea Ş (1964) Die innerartliche Gliederung der Zauneidechse (*Lacerta agilis*) in Rumänien (Reptilia, Lacertidae). Senckenbergiana biologica 45: 469-489.

Georgescu M (1991) Contribuţii la cunoaşterea ofidienilor din zona Târgovişte. Natura 1: 59-61.

Gherghel I, Strugariu A (2007) Antropogenic impact upon the herpetofauna and the lake system from the future natural reserve from “Făurei Swamp”(Neamţ County, Romania). Analele Ştiinţifice ale Universităţii „Al I Cuza” Iaşi, seria Biologie animală 53: 175-179.

Gherghel I, Strugariu A, Ghiurcă D, Roşu S, Huţuleac-Volosciuc MV (2007) The composition and distribution of the herpetofauna from the Valea Neagra river basin (Neamţ County, Romania. Herpetologica 1: 70-76.

Gherghel I, Strugariu A, Glăvan T (2007) *Eremias arguta deserti* (Reptilia: Lacertidae) is not extinct from Romanian Moldavia. North-Western Journal of Zoology 3: 115-120.

Gherghel I, Strugariu A, Pricop E, Zamfirescu SR (2008) The Northern Goşmani Mountains (Romania): An Important Herpetofaunal Area requiring urgent protection. Herpetologica Romanica 2: 51-54.

Gherghel I, Strugariu A, Săhlean CT, Zamfirescu O (2009) Anthropogenic impact or anthropogenic accommodation? Distribution range expansion of the common wall lizard (*Podarcis muralis*) by means of artificial habitats in the north-eastern limits of its distribution range. Acta Herpetologica 4: 183-189.

Ghira I (1997) Herpetofauna of Crişul Repede/Sebes Körös and Barcău/Berettyó river basins. În Sárkány-Kiss A, Hamar J (Eds) TISCIA Monograph series: The Criş/Körös rivers' Valleys. Department of Ecology, University of Szeged, Szeged-Arad, 353-361 pp.

Ghira I, Ghile P (1997) The herpetofauna of the River Someş/Szamos basin. În Sárkány-Kiss A, Hamar J (Eds) TISCIA monograph series: The Someş/Szamos River Valley. Department of Ecology, University of Szeged, Arad-Szeged, 311-317 pp.

Ghira I, Venczel M, Covaciu-Marcov SD, Mara G, Ghile P, Hartel T, Török Z, Farkas L, Rácz T, Farkas Z, Brad T (2002) Mapping of Transylvanian herpetofauna. Nymphaea Folia naturae Bihariae 29: 145-201.

Ghiurcă D, Gherghel I (2008) Aspects concerning the herpetofauna in the city of Bacău (România): urban and periurban environments. Herpetologica Romanica 2: 13-19.

Ghiurcă D, Gherghel I, Roşu G (2009) Contribution to knowledge of the distribution of herpetofauna in Tarcău Mountains (Romania). AES Bioflux 1: 73-79.

Ghiurcă D, Munteanu A, Feneru F (2003) Some herpetological observations in Piatra Craiului National Park. Research in Piatra Craiului National Park 1: 273-274.

Ghiurcă D, Rang G, Roşu S (2006) Preliminary data concerning the herpetofauna in Bacău county. Studii şi Cercetări Ştiinţifice Universitatea din Bacău Seria Biologie 11: 91-98.

Ghiurcă D, Roşu S, Gherghel I (2005) Preliminary data concerning the herpetofauna in Neamţ County (Romania). Analele Universităţii din Oradea, Fascicula Biologie 12: 53-62.

Halpern B, Péchy T, Kiss JB (2002) Scientific research on two isolated *Vipera ursinii moldavica* populations of the Romanian Black Sea coast: preliminary results. Cercetări Marine 34: 301-311.

Haranth GD (1982) *Testudo graeca*, o specie în pericol. Studii şi comunicări Societatea de Ştiinţe Biologice din RSR Filiala Reghin 1: 397-404.

Iana S (1970) Noutăţi faunistice în ecosistemele Dobrogei de sud. Studii şi Comunicări Ocrotirea Naturii Suceava 1: 251-256.

Iftime A (2001) Observations on the amphibians and reptiles of the National Parks Semenic-Cheile Caraşului and Cheile Nerei-Beuşniţa (Romania). Travaux du Muséum National d'Histoire Naturelle "Grigore Antipa" 43: 323-332.

Iftime A (2002) *Testudo hermanni* Gmelin, 1789 in Dobroudja (SE Romania), with comments on conservation. Herpetozoa 15: 183-186.

Iftime A (2003) Contribution to the knowledge of the ichtiofauna and herpetofauna of Piatra Craiului National Park and its surrounding areas. Research in Piatra Craiului National Park 1: 267-272.

Iftime A (2005) Herpetological observations in the Danube Floodplain sector in the Giurgiu county (Romania). Travaux du Muséum National d'Histoire Naturelle "Grigore Antipa" 48: 339-348.

Iftime A (2005) New observations on the herpetofauna from Domogled-Valea Cernei National Park and Porţile de Fier Natural Park (Romania). Travaux du Muséum National d'Histoire Naturelle "Grigore Antipa" 48: 327-337.

Iftime A (2005) Notes on the amphibians and reptiles in the region of Vidraru dam lake (southern cline of the Făgăraş Massif, Romania). Travaux du Muséum National d'Histoire Naturelle "Grigore Antipa" 48: 317-326.

Iftime A, Gherghel I, Ghiurcă D (2008) Contribution to the knowledge on the herpetofauna of Bacău county (Romania). Travaux du Muséum National d'Histoire Naturelle "Grigore Antipa" 51: 243-253.

Iftime A, Iftime O (2006) Herpetofauna masivelor forestiere continentale din sud-vestul Dobrogei. Situaţia actuală şi importanţa acesteia în conservarea habitatelor naturale. Delta Dunării-Studii şi cercetări de ştiinţele naturii şi muzeologie 3: 141-152.

Iftime A, Iftime O (2007) Some records of the herpetofauna of the Danube floodplain in the Balta Ialomiței area (Romania). Travaux du Muséum National d'Histoire Naturelle "Grigore Antipa" 50: 273-281.

Iftime A, Iftime O (2008) Observations on the herpetofauna of the Giurgiu county (Romania). Travaux du Muséum National d'Histoire Naturelle "Grigore Antipa" 51: 209–218.

Iftime A, Iftime O (2010) Contributions to the knowledge of the herpetofauna of the Eastern Jiu and Upper Lotru drainage basins (Southern Carpathians, Romania). Travaux du Muséum National d'Histoire Naturelle "Grigore Antipa" 53: 273-286.

Iftime A, Iftime O (2011) Note on the Herpetofauna of the Vâlcan Mountains and their Foothills (Southern Carpathians, Romania). Travaux du Muséum National d'Histoire Naturelle "Grigore Antipa" 54: 513-521.

Iftime A, Petrescu AM, Iftime O (2008) Observations on the herpetofauna of the Mehedinţi karstic plateau (Mehedinţi and Gorj counties, Romania). Travaux du Muséum National d'Histoire Naturelle "Grigore Antipa" 51: 219-230.

Ion I, Zamfirescu SR, Zamfirescu O, Găucan D (2006) Observations on reptile populations from the zone Probota-Perieni (Iaşi county). Analele Ştiinţifice ale Universităţii „Al I Cuza” Iaşi, seria Biologie animală 52: 197-204.

Ionescu V, Miron I, Munteanu D, Simionescu V (1968) Vertebrate din bazinul montan al Bistriţei. Lucrările staţiunii de cercetări biologice, geologice şi geografice „Stejarul”, Pângaraţi 1: 375-437.

Iordache I, Zamfirescu SR, Antonesei D (2005/2006) Preliminary observations on snake populations from Poiana cu Schit-Bârnova Forest (Iaşi County). Studii şi cercetări de biologie Universitatea din Bacău 11: 99-103.

Iordache I, Zamfirescu SR, Zamfirescu O, Găucan O (2006) Observations on reptile populations from the zone Probota-Perieni (Iaşi county). Analele Ştiinţifice ale Universităţii „Al I Cuza” Iaşi, seria Biologie animală 52: 197-204.

Işfan T (1972) Contribuţii la studiul paraziţilor intestinali la *Testudo graeca* L. Studii şi Cercetări Biologie Seria Zoologie 54: 21-34.

Jessat M (1998) Herpetologische Notizen aus Rumänien (1992-1997). Mauritiana 16: 598-600.

Kiriţescu C (1901) Contributions a l'etude de la faune herpetologique de Roumanie. Premiere partie. Sauriens et ophidiens. Buletinul Societăţii de Ştiinţe 10: 303-328.

Krecsák L, Hartel T (2001) Újabb adatok a pannon gyik (*Ablepharus kitaibelii*) bánsági és olténiai (Romániai) elterjedéséhez. Acta Hargitensia 8: 111-113.

Krecsák L, Sike T, Sós T (2004) Distribution of the herpetofauna in the Lotrioara river basin, Sibiu district (Romania). Travaux du Muséum National d'Histoire Naturelle "Grigore Antipa" 47: 285-295.

Krecsák L, Zamfirescu SR (2008) *Vipera (Acridophaga) ursinii* in Romania: historical and present distribution. North-Western Journal of Zoology 4: 339-359.

Lazăr V, Covaciu-Marcov SD, Sas I, Pusta C, Kovács EH (2005) The herpetofauna in the district of Dolj (Romania). Analele Ştiinţifice ale Universităţii „Al I Cuza” Iaşi, seria Biologie animală 51: 169-178.

Méhely L (1918) Fauna Regni Hungariae. Regia Societas Scientiarum Naturalium Hungarica, Budapest, Hungary, 1006 pp.

Mertens R (1957) Viaţa animalelor din Delta Dunării. Amfibii şi reptile. Natura 9: 80-83

Micluţă H (1970) Note faunistice herpetologice din Judeţul Maramureş. Buletinul Ştiinţific al Institutului Pedagogic Baia Mare, Seria B (Biologie Fizica Chimie Matematică) 2: 39-42.

Mihalca AD, Achelăriţei D, Popescu P (2002) Haemoparasites of the genus *Haemogregarina* in european pond turtles (*Emys orbicularis*) from Drăgăşani, Vâlcea county, Romania. Scientia Parasitologica 2: 22-27.

Oţel V (1992) Investigaţii herpetologice în rezervaţia Biosferei Delta Dunării (RBDD) în anul 1991. Analele Ştiinţifice ale Institutului Delta Dunării 1: 159-162.

Oţel V (1997) Investigaţii herpetologice în zona munţilor Măcin şi podişul Babadagului. Analele Ştiinţifice ale Institutului Delta Dunării 6: 71-77.

Paucă-Comănescu M, Dihoru G, Onete M, Vasiliu-Oromulu L, Falcă M, Honciuc V, Stănescu M, Purice D, Matei B (2004) The diversity of some alluvial shrubland flora and fauna in the Neajlov Floodplain Proceedings of the Institute of Biology 6: 105-118.

Pătroescu M, Chincea I, Rozylowicz L, Sorescu C, Eds., (2007) Pădurile cu pin negru de Banat Natura 2000 site. Editura Brumar, Timişoara, 353 pp.

Petrescu M, Dinu C, Radu A, Cuzic V (2003) The monitoring of the fluvial lakes from southwestern Dobrogea. Studii şi cercetări de biologie Universitatea din Bacău 8: 193-197.

Radu D, Cociu M (1974) Un caz de bicefalie la vipera cu corn *Vipera a. ammodytes* (L.). Studii şi Cercetări Biologie Seria Zoologie 26: 89-91.

Rădulescu I (1961) Contribuţii la cunoaşterea helminţilor broaştei ţestoase de apă *Emys orbicularis* L. din R.P.R. Studii şi Cercetări Biologie Seria Biologie Animală 13: 325-334.

Rozylowicz L (2008) Metode de analiză a distribuţiei areal-geografice a ţestoasei lui Hermann *(Testudo hermanni* Gmelin, 1789) în România. Studiu de caz: Parcul Natural Porţile de Fier. Editura Universităţii din Bucureşti, 169 pp.

Rozylowicz L, Tetelea CD, Popescu VD (2003) Assessing the distribution of Hermann’s tortoise (*Testudo hermanni boettgeri* Mojsisovics, 1888) in the Iron Gates Natural Park, Romania. În: Pătroescu M (Ed) Proceedings of the International Conference on Environmental Research and Assessment. Ars Docendi, Bucharest, 355-366 pp.

Săhlean CT, Meşter LE, Crăciun N (2010) First distribution record for the large whip snake *(Dolichophis caspius* Gmelin, 1789) in the county of Teleorman (Islaz, Romania). Biharean Biologist 4: 181-183.

Săhlean CT, Strugariu A, Zamfirescu SR, Pavel AG, Puşcaşu CM, Gherghel I (2008) A herpetological hotspot in peril: Anthropogenic impact upon the amphibian and reptile populations from the Băile Herculane tourist resort, Romania. Herpetologica Romanica 2: 37-46.

Sas I, Cicort-Lucaciu AS (2012) Some data upon the presence of *Coronella austriaca* (Reptilia) in Carei Plain natural protected area, Romania. Herpetologica Romanica 6: 69-74.

Sas I, Covaciu-Marcov SD, Lucaciu AC, Kovacs EH, Peter V (2004) Studiul variaţiilor fenotipice a unor populaţii de *Zootoca vivipara* din Munţii Apuseni. Oltenia, Studii şi Comunicări, Ştiinţele Naturii 20: 273-279.

Schlüter U (2005) Die herpetofauna des Comorova-Waldes in Rumänien. Elaphe 13: 57-62.

Schlüter U (2005) Die smaragdeidechsen der Dobrudscha. Die Eidechse 16: 46-61.

Sós T (2005) Note preliminare privind distribuţia spaţială a herpetofaunei de pe Culmea Pricopanului din Parcul Naţional Munţii Măcin. Migrans 7: 8-10.

Sós T (2007) Notes on distribution and current status of herpetofauna in the northern area of Braşov County (Romania). North-Western Journal of Zoology 3: 34-52.

Sós T (2011) În obiectiv: Ţestoasa de apă europeană, *Emys orbicularis*. Asociaţia Ecouri Verzi, Cluj-Napoca, 110 pp.

Sós T (2013) Conservation activities for European pond turtles (*Emys orbicularis*) in Romania. Herpetological Notes 5: 147-148

Sós T, Dároczi S (2008) Date suplimentare ale distribuţiei herpetofaunei în Dobrogea. Migrans 10: 2-5.

Sos T, Ghira I, Hegyeli Zs. (2013) New distribution data and conservation status of *Vipera ursinii rakosiensis* (Méhely, 1893) in Transylvania, Romania. 17th European Congress of Herpetology and SEH Ordinary General Meeting, Veszprém (Hungary), 22-27 August 2013, 292.

Sós T, Kecskés A, Hegyeli Z, Marosi B (2012) New data on the distribution of *Darevskia pontica* (Lantz and Cyrén, 1919) (Reptilia: Lacertidae) in Romania: filling a significant gap. Acta Herpetologica 7: 175-180.

Sós T, Szatmári B (2005) Săpăturile arheologice-capcane pentru amfibieni la Roşia Montana. Migrans 7: 8-9.

Stroescu D (1982) Contribuţii la studiul reptilelor din zona Porţile de Fier II. În: Conservarea naturii pe baze ecologice Studii Cercetări Drobeta-Turnu Severin. Drobeta-Turnu Severin, 181-184 pp.

Strugariu A, Butnaru A, Gherghel I, Săhlean CT (2008) First record of the Smooth Snake (*Coronella austriaca* Laurentus, 1768) in Botoşani County (Romania). Biharean Biologist 2: 64-67.

Strugariu A, Gherghel I (2008) A preliminary report on the composition and distribution of the herpetofauna in the Lower Prut River Basin (Romania). North-Western Journal of Zoology 4: 49-69.

Strugariu A, Gherghel I, Ghira I, Covaciu-Marcov SD, Mebert K (2011) Distribution, habitat preferences and conservation of the dice snake (*Natrix tessellata*) in Romania. Mertensiella 18: 272-287.

Strugariu A, Gherghel I, Huţuleac-Volosciuc MV, Puşcaşu CM (2007) Preliminary aspects concerning the herpetofauna from urban and peri-urban environments from North-Eastern Romania: a case study in the city of Suceava. Herpetologica Romanica 1: 53-61.

Strugariu A, Gherghel I, Nicoară A, Huţuleac-Volosciuc MV, Moraru V, Mizeruş A (2009) A rapid survey of the herpetological fauna from Vaslui County (Romania) with the first record of the slow-worm (*Anguis fragilis*) in the region. Herpetologica Romanica 3: 25-30.

Strugariu A, Gherghel I, Zamfirescu SR, Săhlean CT (2008) Spatial distribution of the herpetofauna from the upper and middle Moldova river basin (Romania). Travaux du Muséum National d’Histoire Naturelle "Grigore Antipa" 51: 231–241.

Strugariu A, Săhlean CT, Volosciuc-Huţuleac MV, Puşcaşu CM (2006) Preliminary data regarding the distribution of reptilian fauna in Suceava County (Romania). North-Western Journal of Zoology 2: 39-43.

Strugariu A, Sós T, Sotek A, Gherghel I, Hegyeli Z (2009) New locality records for the adder (*Vipera berus*) in the Carpathian Corner, Romania. AES Bioflux 1: 99-103.

Strugariu A, Zamfirescu SR, Gherghel I (2009) First record of the adder (*Vipera berus berus*) in Argeş County (Southern Romania). Biharean Biologist 3: 163-166.

Strugariu A, Zamfirescu SR, Nicoară A, Gherghel I, Sas I, Puşcaşu CM, Bugeac T (2008) Preliminary data regarding the distribution and status of the herpetofauna in Iaşi County (Romania). North-Western Journal of Zoology 4: S1-S23.

Stugren B (1961) Reptilele grindurilor fluvio-maritime din Delta Dunării (Notă preliminară). Studia Universitatis Babeş-Bolyai Biologie 2: 179-185.

Stugren B (1966) Note faunistice herpetologice din Republica Socialistă România. Studii şi cercetări de biologie Seria Zoologie 18: 103-108.

Stugren B, Ghira I, Kiss JB (1981) Cercetări asupra productivităţii secundare la şopîrla de cîmp, *Lacerta agilis* din Transilvania. Studii şi Comunicări Ocrotirea Naturii Suceava: 277-286.

Ţibu PL, Strugariu A (2007) A new record for the Blotched snake *Elaphe sauromates* (Reptilia: Colubridae) in Romania. North-Western Journal of Zoology 3: 62-65.

Török Z (1996) The protection of the herpetofauna in the Danube Delta. Rezumatele lucrărilor Simpozionului jubiliar "Rezervaţia naturală Codrii-25 de ani Realizări, probleme, perspective" Lozova, Republica Moldova: 124-126.

Török Z (1997) Data on the actual status of the amphibian and reptile populations of the Someş river catchment area (Romania). Studii şi Cercetări Ştiinţifice Universitatea din Bacău Seria Biologie 2: 227-232.

Török Z (1997) Data on the amphibians and reptiles from the Lăpuş river catchment area (Romania). Travaux du Muséum National d'Histoire Naturelle "Grigore Antipa" 39: 197-207.

Török Z (1997) Data on the ecology of amphibians and reptiles from the sandy areas of the Razim-Sinoe Lagoonary System (Romania). Travaux du Muséum National d'Histoire Naturelle "Grigore Antipa" 37: 297-303.

Török Z (1997) Herpetofauna bazinului Baia Mare. Analele Universităţii "Ovidius" Constanţa, Seria Biologie-Ecologie 1: 153-158.

Török Z (1998) Nişe ecologice spaţiale ale herpetofaunei de pe grindurile fluvio-maritime din complexul lagunar Razim-Sinoe (România). Acta Oecologica-Studii şi Comunicări de Ecologie şi Protecţia Mediului 5: 59-63.

Török Z (1998) A quantitative approach to the road mortality in the herpetofauna from Grindul Chituc (Romania). Analele Ştiinţifice ale Institutului Delta Dunării 6: 159-166.

Török Z (1999) Contributions to the knowledge of the distribution of Sand Lizard *(Lacerta agilis euxinica* Fuhn and Vancea 1964) in south-eastern Romania. Analele Ştiinţifice ale Institutului Delta Dunării 7: 498-500.

Török Z (1999) Data on the distribution of Amphibians and Reptiles in Gutâi and Igniş Mountains. Studii şi Cercetări Ştiinţifice Universitatea din Bacău Seria Biologie 4: 113-118.

Török Z (1999) Note privind distribuţia spaţială a herpetofaunei în zona Culmii Pricopanului (Jud. Tulcea, România). Acta Oecologica-Studii şi Comunicări de Ecologie şi Protecţia Mediului 6: 57-62.

Török Z (1999) Zonele umede din nord-vestul Dobrogei. Probleme de Ecologie Teoretica si Aplicata din Romania-Directii Actuale. Editura Aves, Tulcea, 16 pp.

Török Z (2000) Date privind corologia şi protecţia amfibienilor şi reptilelor din Munţii Igniş. Satu-Mare, Studii şi Comunicări, Seria Ştiinţele Naturii 1: 160-170.

Török Z (2001) Herpetological investigations in the lower Danube area (Calafat-Călăraşi sector). Studii şi Cercetări Ştiinţifice Universitatea din Bacău Seria Biologie 6: 115-119.

Török Z (2002) Grindul Chituc. Probleme de Ecologie Teoretica si Aplicata din Romania-Directii Actuale. Editura Aves, Tulcea, 16 pp.

Török Z (2003) Potential management plan for conservation of *Vipera ursinii* from the Danube Delta Biosphere Reserve (Romania). Analele Ştiinţifice ale Institutului Delta Dunării 9: 174-184.

Török Z (2004) Data on the actual status of lake Plopu-Beibugeac (Tulcea county, Romania). Analele Ştiinţifice ale Institutului Delta Dunării 10: 71-80.

Török Z (2004) Herpetological investigations in the Danube Delta Biosphere Reserve (Romania) in 2003. Analele Ştiinţifice ale Institutului Delta Dunării 10: 81-83.

Török Z (2006) GIS technique used for managing data on potential Natura 2000 sites. Case study: areas inhabited by *Elaphe quatuorlineata*. Analele Ştiinţifice ale Institutului Delta Dunării 12: 201-210.

Török Z (2008) Taxonomia şi ecologia populaţiilor de şopârle (Reptilia: Lacertidae) din Dobrogea de Nord. Bucureşti: PhD thesis. Universitatea din Bucureşti.

Tudor M, Crăciun N, Burlacu L (2004) Preliminary report on herpetofauna of the becoming National Parc „Jiului Gorge”. Oltenia, Studii şi Comunicări, Ştiinţele Naturii 20: 269-272.

Zamfirescu SR (1999) The mapping of herpetological fauna from the left side of Bicaz accumulation lake. Analele Ştiinţifice ale Universităţii „Al I Cuza” Iaşi, seria Biologie animală 44/45: 143-146.

Zamfirescu SR, Strugariu A, Gherghel I, Zamfirescu O (2010) Sfântu Gheorghe (Tulcea, Romania): an important herpetological area. Analele Ştiinţifice ale Universităţii „Al I Cuza” Iaşi, seria Biologie animală 56: 119-128.

Zamfirescu SR, Zamfirescu O, Strugariu A, Gherghel I (2009) Herpetofauna of the meadows from the Site of Community Interest “The forest and the meadows from Mârzeşti” (Iaşi, Romania) and notes on habitats. Analele Ştiinţifice ale Universităţii „Al I Cuza” Iaşi, seria Biologie animală 55: 155-163.

Zinenko O, Ţurcanu V, Strugariu A (2010) Distribution and morphological variation of *Vipera berus nikolskii* Vedmederja, Grubant et Rudaeva, 1986 in Western Ukraine, The Republic of Moldova and Romania. Amphibia-Reptilia 31: 51-67.

Zinke O, Hielscher K (1990) Nachweis der Westlichen Sandboa *(Eryx jaculus turcicus* [Olivier]) in Rumänien (Reptilia, Serpentes: Boidae). Faunistische Abhandlungen Staatliches Museum für Tierkunde Dresden 17: 191-192.
