# Supplementary material for: Diversity and distribution of reptiles in Romania
Source: Zookeys. 2013 Oct 8;(341):49–76. doi: 10.3897/zookeys.341.5502 (PMC3800809; doi:10.3897/zookeys.341.5502)

**Appendix 2**

**The reptiles occurrence records within Romania per time intervals (1961 represented the publishing year of the last country wide assessment).**


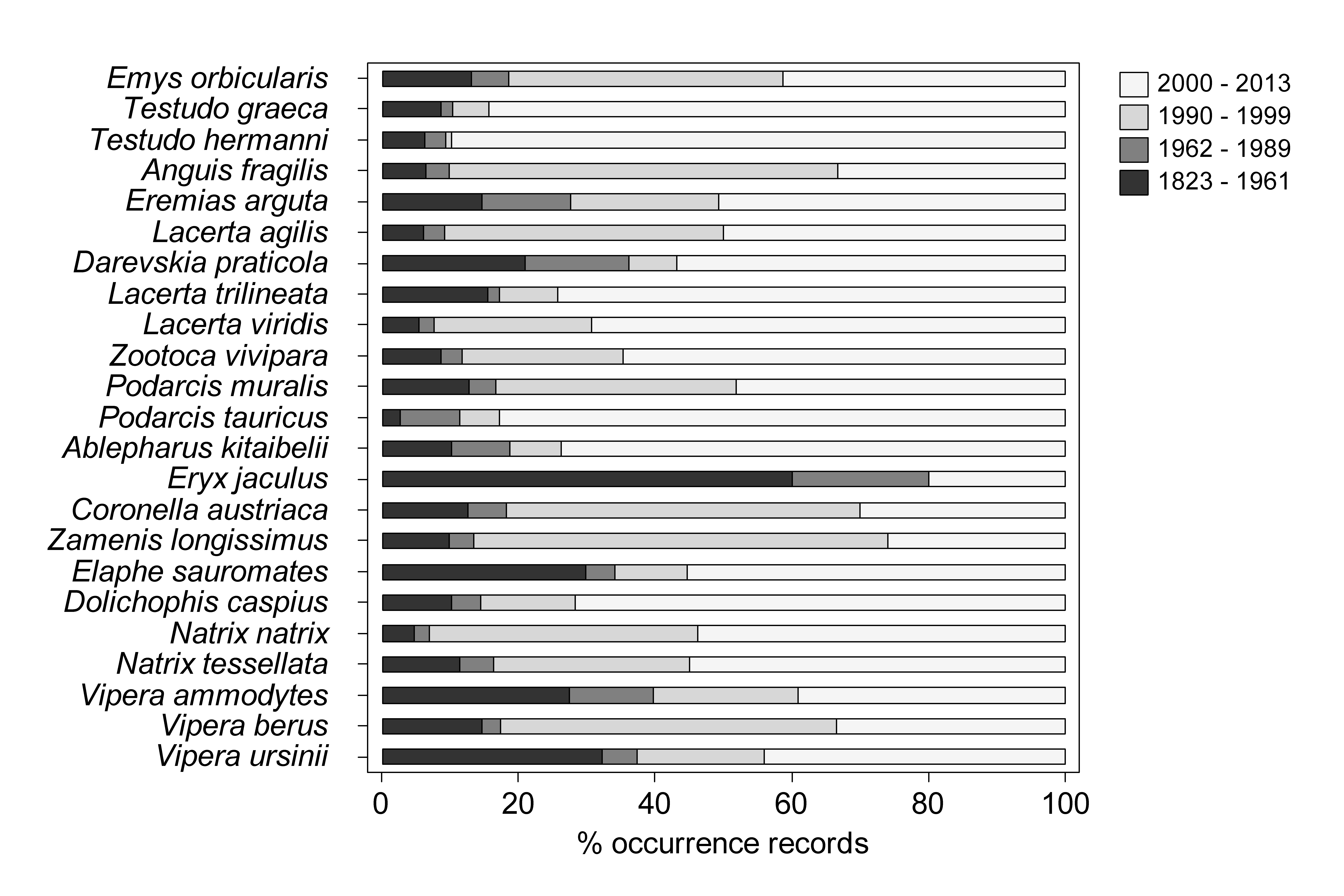

Supplement: Supplementary file 2 — The reptiles occurrence records within Romania per time intervals (1961 represented the publishing year of the last country wide assessment). (doi: 10.3897/zookeys.341.5502.app2) File format: Microsoft Word file (doc). [file ZooKeys-341-049-s002.doc]
