# Supplementary material for: Diversity and distribution of reptiles in Romania
Source: Zookeys. 2013 Oct 8;(341):49–76. doi: 10.3897/zookeys.341.5502 (PMC3800809; doi:10.3897/zookeys.341.5502)

**Appendix 3**

**The reptile species richness at a 10 × 10 km grid resolution within Romania (SCI = Natura 2000 Sites of Community Importance).**


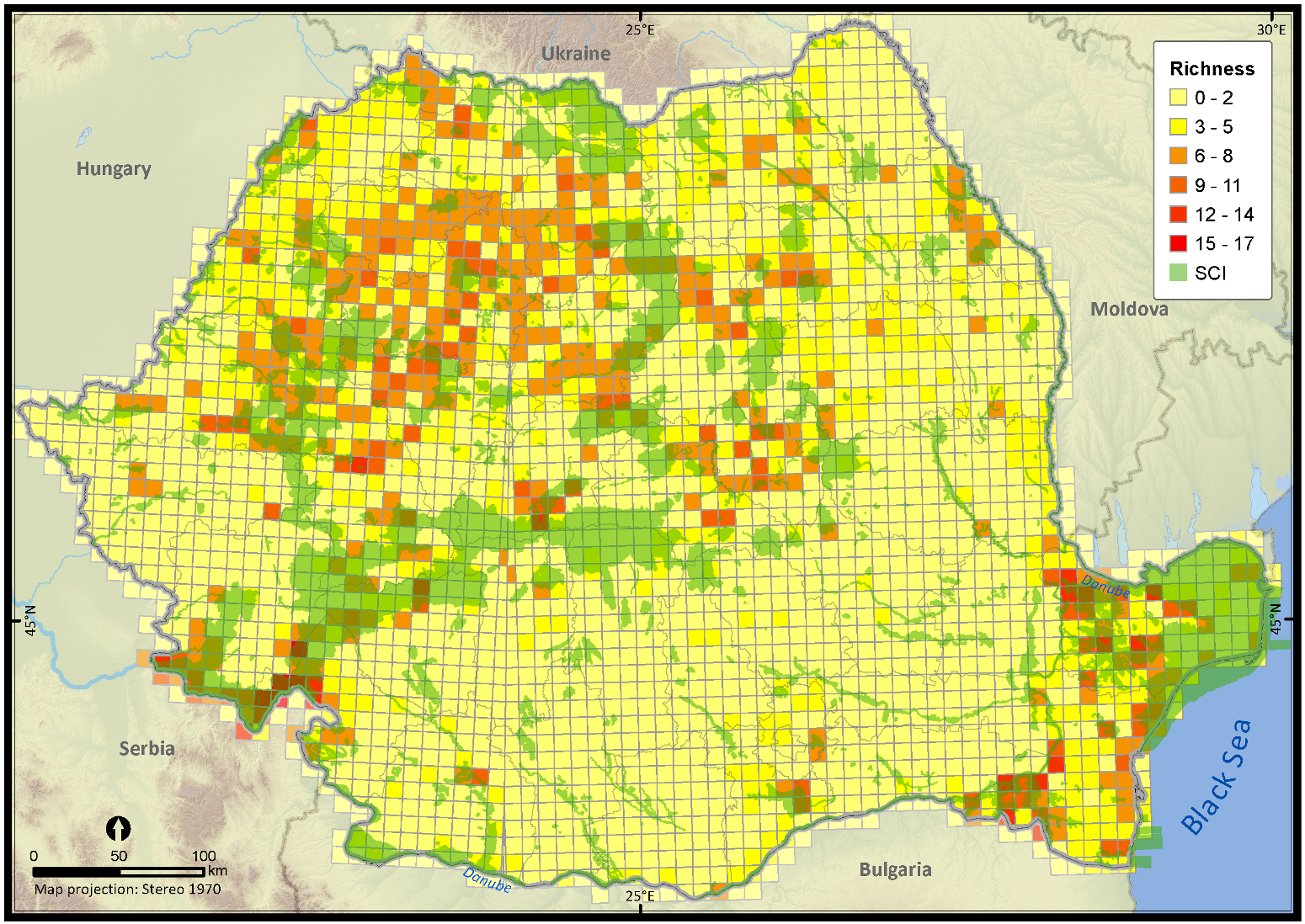

Supplement: Supplementary file 3 — The reptile species richness at a 10 × 10 km grid resolution within Romania (SCI = Natura 2000 Sites of Community Importance). (doi: 10.3897/zookeys.341.5502.app3) File format: Microsoft Word file (doc). [file ZooKeys-341-049-s003.doc]
